# Changes in Evolutionary Developmental Control Points in the Amniote Limb May Explain Hyperphalangy

**DOI:** 10.1093/molbev/msaf113

**Published:** 2025-06-09

**Authors:** Merijn A G de Bakker, Luthfi Nurhidayat, Alisha Kiran Dijkerman, Wing Yu Chung, Elena C Oudesluys, Kaylah de Jager, Joost Willemse, Michael K Richardson

**Affiliations:** Animal Science & Health, Institute of Biology Leiden (IBL), Leiden University, Leiden 2333BE, the Netherlands; Animal Science & Health, Institute of Biology Leiden (IBL), Leiden University, Leiden 2333BE, the Netherlands; Faculty of Biology, Universitas Gadjah Mada, Yogyakarta 55281, Indonesia; Animal Science & Health, Institute of Biology Leiden (IBL), Leiden University, Leiden 2333BE, the Netherlands; Animal Science & Health, Institute of Biology Leiden (IBL), Leiden University, Leiden 2333BE, the Netherlands; Animal Science & Health, Institute of Biology Leiden (IBL), Leiden University, Leiden 2333BE, the Netherlands; Animal Science & Health, Institute of Biology Leiden (IBL), Leiden University, Leiden 2333BE, the Netherlands; Animal Science & Health, Institute of Biology Leiden (IBL), Leiden University, Leiden 2333BE, the Netherlands; Animal Science & Health, Institute of Biology Leiden (IBL), Leiden University, Leiden 2333BE, the Netherlands

**Keywords:** evo-devo, limb, evolution, amniote, hyperphalangy, development

## Abstract

Amniotes show a great diversity of limb phenotypes, including limbs specialized for running, flying, swimming, and digging. Here, we have examined how this diversity is generated during limb development in 13 species using transcriptomics and in situ hybridization. The selected species show evolutionary changes in the number of phalanges and/or loss of claws. We first looked at genes that show cyclical expression during digit development. Significantly, we find that *Gdf5* cycles more rapidly in digits developing more phalanges. We identified two novel cyclically expressed genes: *Ackr3* and *Wnt9a*. We also identified a transition point at which phalanx formation stops and claw development begins. We found that this transition point is marked by the downregulation of multiple developmental genes in the phalanx-forming region, and upregulation of claw-related genes. The timing of this transition is conserved, taking place at the same developmental stage in all digits of all species examined—except in the clawless digits of the Chinese soft-shelled turtle, the crocodilians, and birds. We suggest a model based on transcriptional heterochrony, in which the frequency of phalanx formation and the timing of the phalanx–claw transition are evolutionary control points open to natural selection on the phenotype. Furthermore, our model suggests that relaxation of developmental constraints on the timing of the phalanx–claw transition allows the digits to develop more phalanges (hyperphalangy). This is seen in some turtles, crocodilians, and dolphins. More broadly, our findings are consistent with the hypothesis that “hotspots” in otherwise conserved developmental pathways may be targets for evolutionary tinkering.

## Introduction

**Fig. 1.**
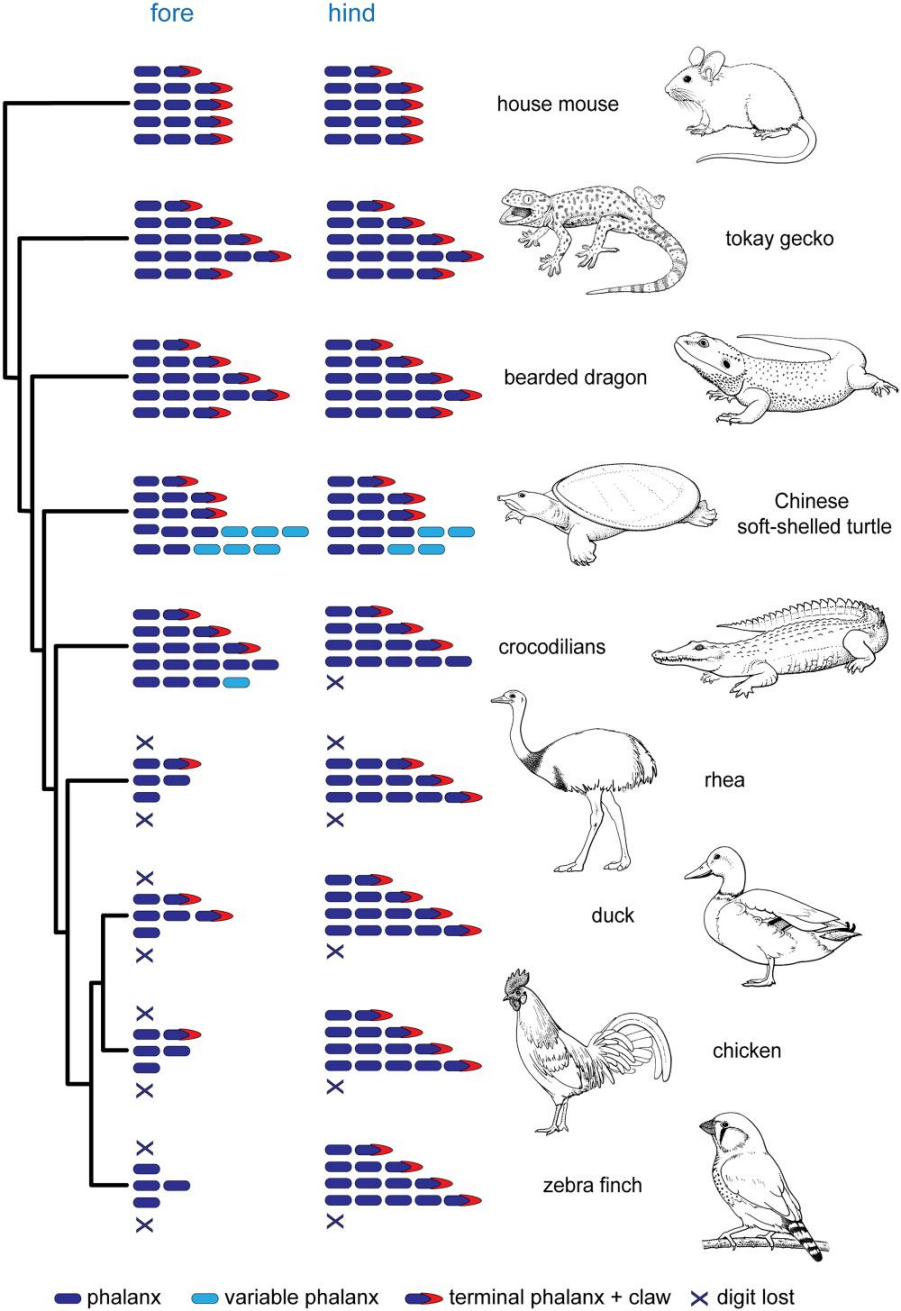
Schematic summary of phenotypic changes in the autopod of selected amniotes. The central bearded dragon (*Pogona vitticeps*) and tokay gecko (*Gekko gecko*) retain the plesiomorphic condition of the autopod (Zaaf et al. 1999; Simoes et al. 2017; de Bakker et al. 2021); all other species studied here show various deviations from that ancestral phenotype. The mouse has the typical mammalian phalangeal formula and has lost one phalanx in digit III and two in digit IV in the forelimb, and in the hindlimb the same except, additionally, one is lost from digit V (Parmenter et al. 2016). In the Chinese soft-shelled turtle (*Pelodiscus sinensis*), digit III has lost one phalanx, while digits IV and V have a variable number of phalanges and have lost the claw (Delfino et al. 2010). In the crocodilians (we used three species; *Caiman latirostris*, *Osteolaemus tetraspis*, and *Crocodylus niloticus*), forelimb digits IV and V have lost the claws, as has hindlimb digit IV, and hindlimb digit V has been completely lost (Reynolds 1897; Müller and Alberch 1990; de Bakker et al. 2013). The greater rhea (*Rhea americana*) forelimb has lost digits I and V, has lost one phalanx from digit II, two phalanges and the claw from digit III, and four phalanges and the claw compared to the primitive condition. In the rhea, hindlimb digits I and V have been completely lost (Fisher 1940; Maxwell and Larsson 2009; de Almeida et al. 2015). The duck (*Anas platyrhynchos*) wing has lost one phalanx from digit II, one phalanx from digit III, and three phalanges and a claw from digit IV (Richardson 2012). The duck hindlimb has lost digit V, but digits I–IV have the full ancestral complement of phalanges and claws (de Bakker et al. 2021). Compared to the duck, the chicken (*Gallus* gallus) has lost one additional phalanx and a claw from wing digit III, while the zebra finch (*Taeniopygia guttata*) has lost a phalanx and claw from wing digit II; both species have a similar hindlimb phenotype as the duck (Richardson 2012 ; de Bakker et al. 2013). The phylogeny is based on Chiari et al. (2012), Green et al. (2014), Jarvis et al. (2014) , and Stein et al. (2015) . Line drawings by Esmée Winkel.

The terminal part of the amniote limb is the autopod (hand or foot). It includes the digits, which are made up of phalanges and often carry a claw. The ancestral autopod of amniotes (mammals + sauropsids [reptiles including birds]) probably had, as *Pogona vitticeps* does now, five clawed digits with a phalangeal formula of 2-3-4-5-3 in digits I–V in the forelimb and 2-3-4-5-4 in the hindlimb (Fig. 1 and references Williston 1925; Romer 1956; Smithson 1989; Sumida 1997; de Bakker et al. 2021; Mann et al. 2021). During the adaptive radiation of amniotes (Sumida and Martin 1996; Brocklehurst and Benson 2021), the ancestral limb and its claws became variously adapted to such niches as swimming, flying, digging, climbing, and moving on land (Fig. 1; see also Hamrick 2001; Ethier et al. 2010; de Bakker et al. 2013; Baeckens et al. 2020 ; Doody et al. 2020; Alibardi 2021). As a result, the autopod shows remarkable variation between species, making it a good model for studying morphological evolution (Delfino et al. 2010).

Various phenotypic changes are associated with the adaptation of the ancestral amniote limb to different niches. For example, digits sometimes lost one or more phalanges while retaining the claw, as in tortoises and most mammals (de Bakker et al. 2013); or they lost phalanges and the claw, as in the digits of the chicken wing (Casanova et al. 2012); or they showed an increase in the number of phalanges and a loss of the claw, as in the posterior digits of soft-shelled turtles (Trionychidae) and crocodilians, and the flipper of cetaceans (whales and dolphins; Richardson and Oelschlager 2002; Fedak and Hall 2004; Cooper et al. 2007). In the Chinese soft-shelled turtle (*Pelodiscus sinensis*) and crocodilians, digits I–III are claw-bearing and are used for walking on land, whereas digits IV and V are slender, clawless “swim-fingers” that support the skin-web used in swimming ([Rabl 1910; Delfino et al. 2010; de Bakker et al. 2021]; note that digit V has been lost in the crocodilian hindlimb). Digits IV and V in the Chinese soft-shelled turtle may develop 2–3 additional phalanges, a condition known as hyperphalangy (Delfino et al. 2010). The American alligator (*Alligator mississippiensis*) also develops one extra phalanx in the clawless forelimb digit V (Müller and Alberch 1990). In birds, the evolution of the wing involved loss of at least one phalanx from all digits, loss of claws from at least one digit, and complete loss of digits I and V (Fig. 1; Richardson 2012; de Bakker et al. 2013, 2021).

Claws are parts of the phenotype that interact physically with the environment in a way that facilitates specialized behavior (e.g. digging and locomotion; Maddin et al. 2009; Thomson and Motani 2023). Among amphibians (Lissamphibia), claws are only seen in clawed toads and a few species of salamander (Maddin et al. 2009 ; Alibardi 2021). Because of the functional importance of the claws, this study includes stages of limb development up to chicken stage 36 when claw development is underway.

Phalanx number, and the presence or absence of claws, are traits determined during embryonic development. Phalanges and synovial joints are specified in the phalanx-forming region (PFR), a growth zone at the tip of the developing digits consisting of mesenchyme capped by part of the former apical ectodermal ridge (AER). The gene *Sox9* is a marker of the PFR (Suzuki et al. 2008; Huang et al. 2016 ; de Bakker et al. 2021).

**Fig. 2.**
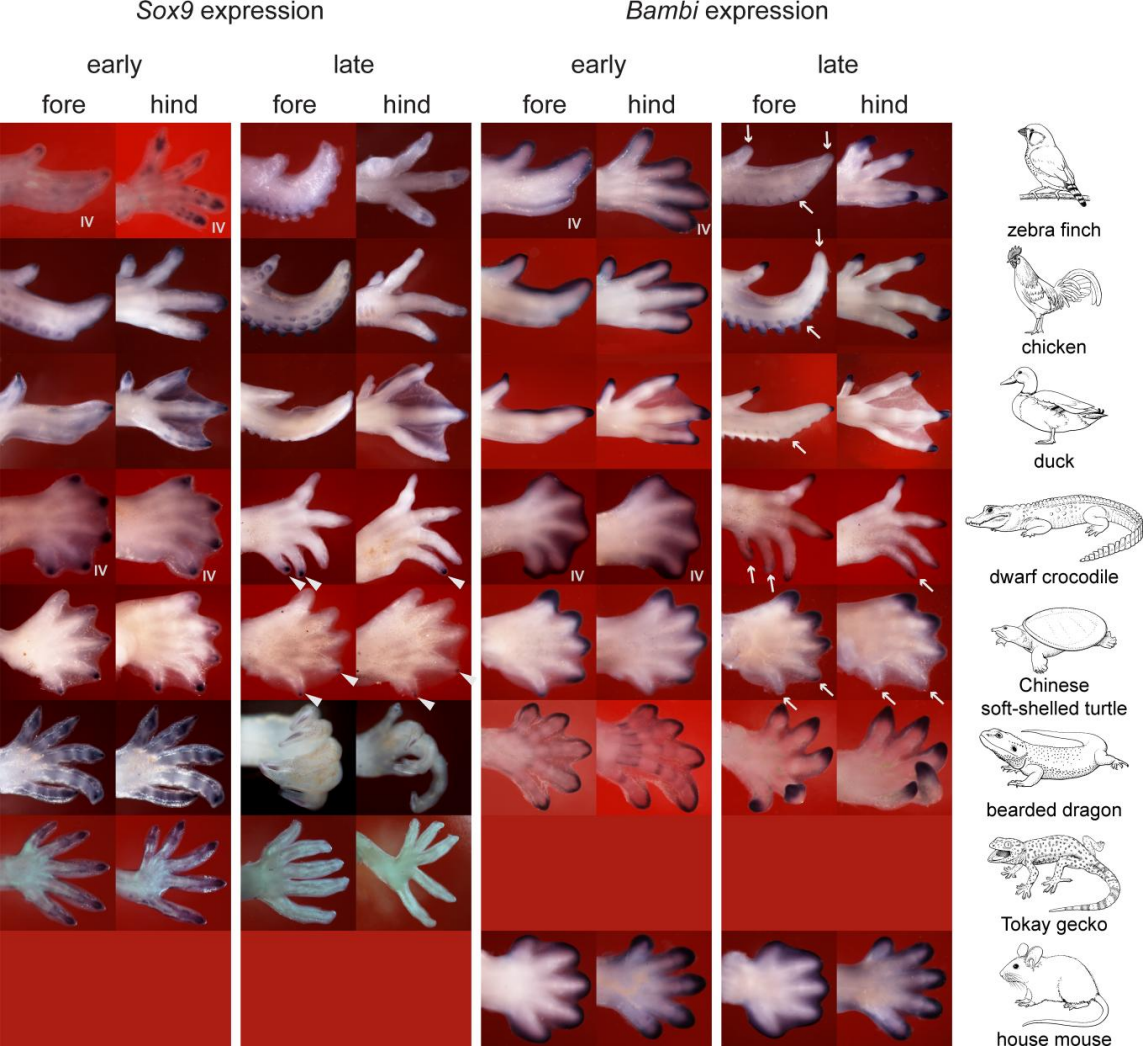
A phalanx–claw transition as shown by *Sox9* and *Bambi* expression in selected amniotes. The species shown are (top to bottom): *Taenopygia guttata*, *Gallus gallus*, *Anas platyrhynchos*, *Osteolaemus tetraspis*, *Pelodiscus sinensis*, *Pogona vitticeps*, *Gekko gecko*, and *Mus musculus*. Anterior is to the top, distal to the right. In the early stage limbs, the digit tips express *Sox9* (in the PFR) but in the late stage, after the phalanx-to-claw transition, *Sox9* is downregulated. The exceptions are the posterior, clawless digits of the dwarf crocodile and turtle (arrowheads) which still express *Sox9* at the late stage. In all species shown, the early *Bambi* expression round the periphery of the digital plate becomes restricted to the digit tips at later stages. However, this restriction of *Bambi* expression fails to occur in clawless digits in the bird wing and turtle and dwarf crocodile limbs (arrowheads, persistent expression of *Sox9*; arrows, down-regulation of *Bambi*, in the same digits and specimens as the photos with arrowheads).

In the chicken, the AER disappears at stages 32 and 33 (Saunders 1977) and claw morphogenesis in the forelimb and hindlimb begins at stage 36 (Hamburger and Hamilton 1951). At this stage, *Bambi* is restricted to the digit tip, in the ectoderm of the PFR, marking the onset of claw development. Before this stage, *Bambi* was expressed in the AER and underlying mesenchyme around the entire margin of the limb (Fig. 2; Grotewold et al. 2001; Casanova et al. 2012). The shift in *Bambi* expression takes place synchronously in all digits in the chicken, mouse and duck at chicken stage 36 (Grotewold et al. 2001; Casanova et al. 2012). Another gene linked to claw development is *Msx1* (Reginelli et al. 1995; Hamrick 2001 ; Bensoussan-Trigano et al. 2011); in the Chinese soft-shelled turtle, *Msx1* and its paralog *Msx2* are expressed at the tips of claw-bearing digits I–III (Fig. 4 in Cordeiro et al. 2020). Interestingly, the claws in the hindlimb of the *Xenopus laevis* (the African clawed toad) develop synchronously (Maddin et al. 2009; Keenan and Beck 2016; Alibardi 2021). The synchronous onset of *Bambi* expression in the digit tips marks the termination of phalanx formation. In Sauropsida (“reptiles” including birds), the digits will have developed two to five phalanges in the period leading up to claw formation. In Mammalia, only two or three phalanges develop before the claw develops (most mammals show a derived phalanx formula of 2-3-3-3-3 [Fig. 1; Parmenter et al. 2016]).

Phalanx development is influenced by many developmental patterning genes including Tgfb1, Fgf, and members of the Wnt family, along with posterior Hox genes, *Noggin*, *Gdf5*, and *Gli3* (Huang et al. 2016). The specification of phalanges may be controlled by an oscillator mechanism (Pascoal et al. 2007; Chinnaiya et al. 2014; Saiz-Lopez et al. 2017). In the axial skeleton of vertebrates, some genes with oscillating expression patterns have been likened to “clocks” whose clock speed or oscillation frequency can be modified by natural selection (Gomez et al. 2008; Vonk and Richardson 2008). *Hes1* (formerly known as *cHairy2*) is a candidate cyclically expressed gene in limb development (Jouve et al. 2000; Pascoal et al. 2007). An alternative model envisages a Turing-like mechanism that generates periodic patterns that specify phalanx development (Raspopovic et al. 2014; Scoones and Hiscock 2020; Grall et al. 2024).

We have examined limb development in 13 amniote species that show evolutionary changes in the number of phalanges and/or loss of claws using transcriptomics and in situ hybridization. We compared them with the ancestral amniote limb phenotype in the bearded dragon (*Pogona vitticeps*; Williston 1925; Romer 1956; Smithson 1989 ; Sumida 1997; de Bakker et al. 2021; Mann et al. 2021). The limbs of all species are staged according to Hamburger and Hamilton for easy comparison (Hamburger and Hamilton 1951). By comparing the development of autopods in species belonging to the same clade but showing diverse phenotypes, we follow the advice of Cuvier who advocated the study of “experiments ready prepared by Nature” (Cuvier 1840, p. 15).

## Results

### Phalanges Are Formed in a Similar Time Window in All Species

**Fig. 3.**
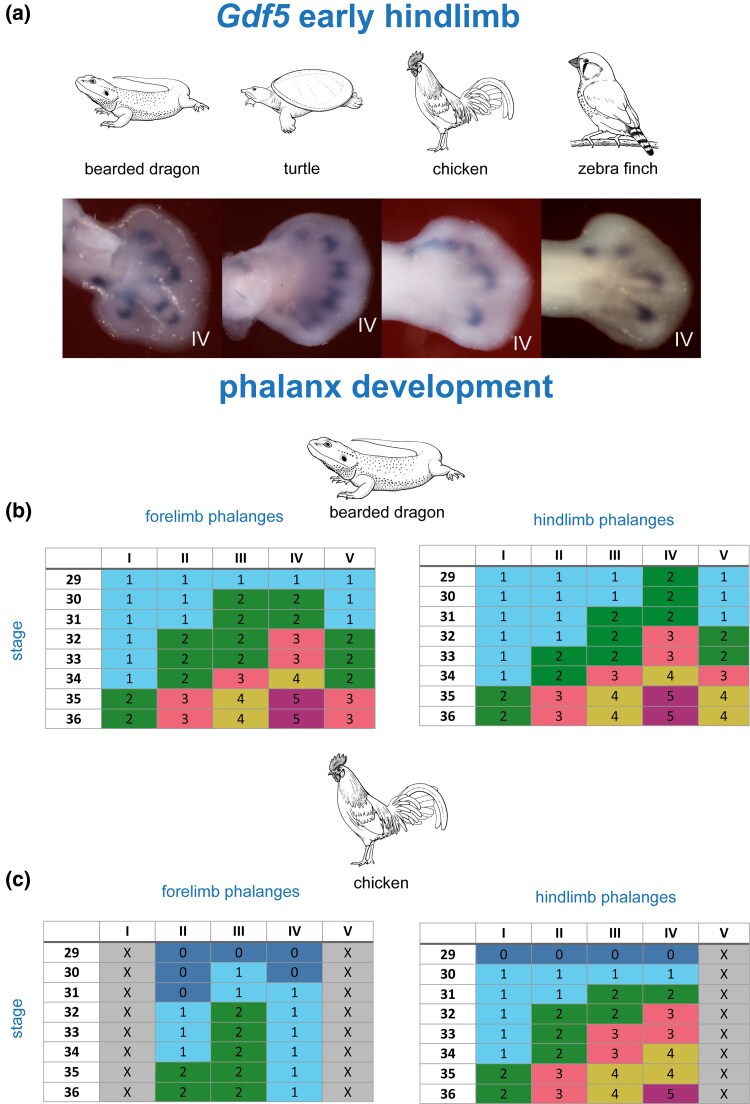
Different numbers of phalanges form with different cyclical patterns—but within the same developmental time-window (stages 29–36). a) Expression of *Gdf5* in chicken stage 28 and 29 hindlimbs of the bearded dragon, Chinese soft-shelled turtle, chicken, and zebra finch. In this early stage, only the metatarsophalangeal joints express *Gdf5*. b and c) Joint development in the bearded dragon and chicken. The data in these tables are derived from the series of whole mounts in supplementary fig. S1, Supplementary Material online. In the top rows, the digits are labeled I–V, from anterior to posterior. The first column of each table indicates the chicken (HH) stages. The developing joints are color-coded and numbered 1–5 from proximal to distal as follows: dark blue, no joint developed; light blue, first joint veloped; green, second joint developed; pink, third joint developed; khaki, fourth joint developed; purple, fifth joint developed. The numbers in the rows correspond to the most recently-formed joint as indicated by *Gdf5* expression. Line drawings by Esmée Winkel.

We find that the onset of phalanx formation is at chicken stage 29 (± 1 stage) in all digits in all species, as indicated by *Gdf5* expression in the metapodial-phalangeal joint (Fig. 2; supplementary fig. S1, Supplementary Material online) The exception to this is the highly-derived avian wing. Thus, in the chicken, the first phalanx of each digit forms over the range of stages 30–32 (Fig. 3c). The offset of phalanx formation is at stage 36 (± 0.5 stages) when *Sox9*, a marker of the PFR, is downregulated; and *Bambi*, a marker of claw development, is upregulated (Fig. 2). In the chicken, we find that in addition to *Sox9*, six other genes expressed in the PFR (*Ackr3*, *Bmpr1b*, *Hes1*, *Hes2*, *Id4*, and *Wnt9a*) are downregulated around stage 36 (supplementary fig. S2, Supplementary Material online). Furthermore, the upregulation at this stage of the claw markers *Msx1* and *Msx2* confirms the *Bambi* results (supplementary fig. S3, Supplementary Material online).

### The Oscillation Frequency of *Gdf5* Expression in a Digit Is Correlated With the Number of Phalanges It Will Develop

**Fig. 4.**
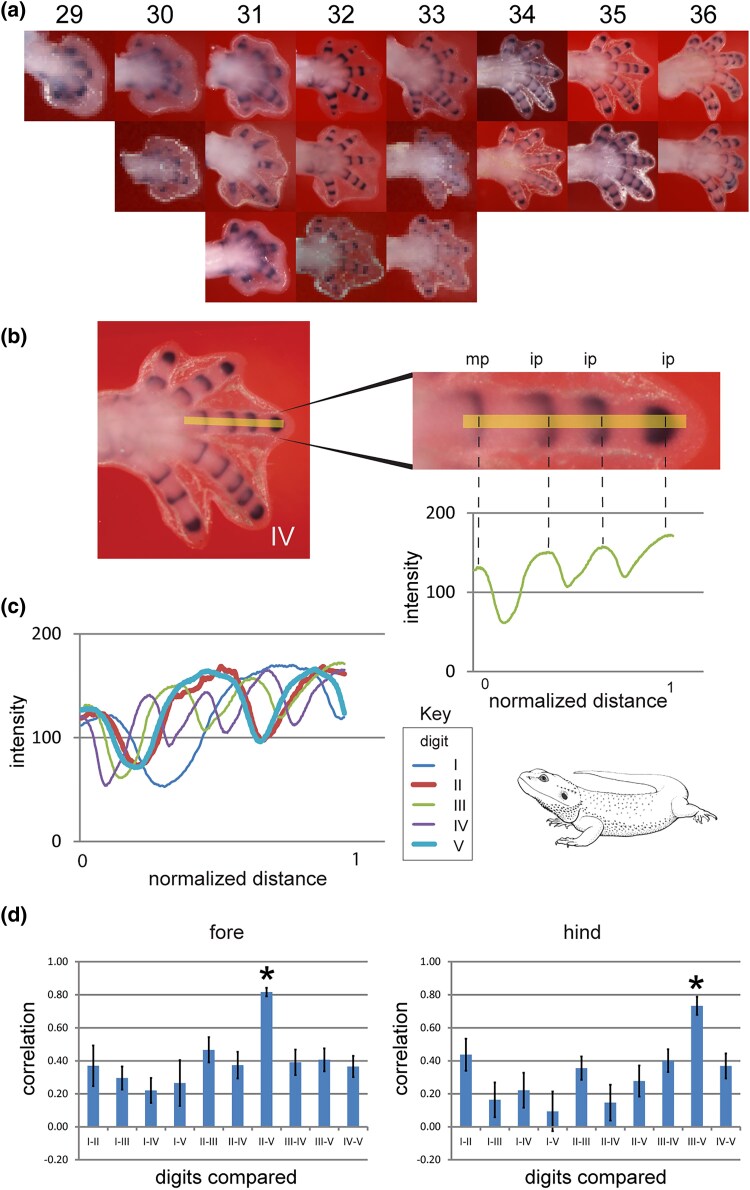
High correlation in the phase of the cyclical expression of *Gdf5* in bearded dragon digits having the same phalanx number. The expression of *Gdf5* starts in the PFR and persists in the developing joints as the digit tip grows out. a) The 18 forelimbs measured for this study. Anterior is to the top, distal to the right; the youngest (stage 29) is on the left; the oldest (stage 36) is on the right. Second and third rows are replicates of the same stages. The hindlimbs were measured in the same way. b) To illustrate our approach, we show here only one example (stage 33, digit III). The expression intensities in this digit were measured by drawing a line from distal to proximal (yellow line). The intensity of of purple color in each pixel along that line was measured. Depending on the size of each digit, we measured between 184 and 1,195 datapoints. Together, these datapoints form the continuous green line shown in the graph below this digit III (staining density against normalized length). c) Graphs of all five digits of the example autopod in b), each digit length normalized for easier comparison. The thicker red and light blue lines are from the isomorphic digits II and V (here isomorphic means having the same number of phalanges in the adult). d) Bar graphs of correlations of intensity measurements of all digits; right side forelimbs and left side hindlimbs. The bar graphs are the accumulation of the correlation coefficients of the 18 samples shown in a) and span therefore the development from 29 to 36 chicken stage. The *Pogona vitticeps* isomorphic forelimb digits II and V and hindlimb digits III and V (asterisks) show the strongest correlation. For further details of the analytical technique, see Material and Methods.

A study (Grall et al. 2024) reported that *Gdf5*, *Noggin*, and *pSmad* show cyclical expression in digits III and IV of the chick and the mouse. We have explored those findings in a broader phylogenetic sample of 13 species (Fig. 1; supplementary table S1, Supplementary Material online). We also explored the cyclical expression of *Gdf5* in more detail in a series of chicken and bearded dragon limbs (Fig. 3). In the bearded dragon, which has the plesiomorphic condition of the autopods (Romer 1956; Wagner and Gauthier 1999; Vargas et al. 2008; Richardson 2012; de Bakker et al. 2013; Xu and Mackem 2013 ; Simoes et al. 2017), forelimb digits II and V have three phalanges, and hindlimb digits III and V have four phalanges. In a model based on cyclically expressed genes, these isomorphic digits should have the same oscillation pattern during development. Our comparison of 18 developing bearded dragon autopods (Figs. 3 and 4) shows a high correlation between the phase of the *Gdf5* expression cycle in forelimb digits II and IV, supporting the hypothesis that the oscillation frequency in a digit is related to its final phalanx number.

### Identification of Novel Cyclically Expressed Genes in the PFR

**Fig. 5.**
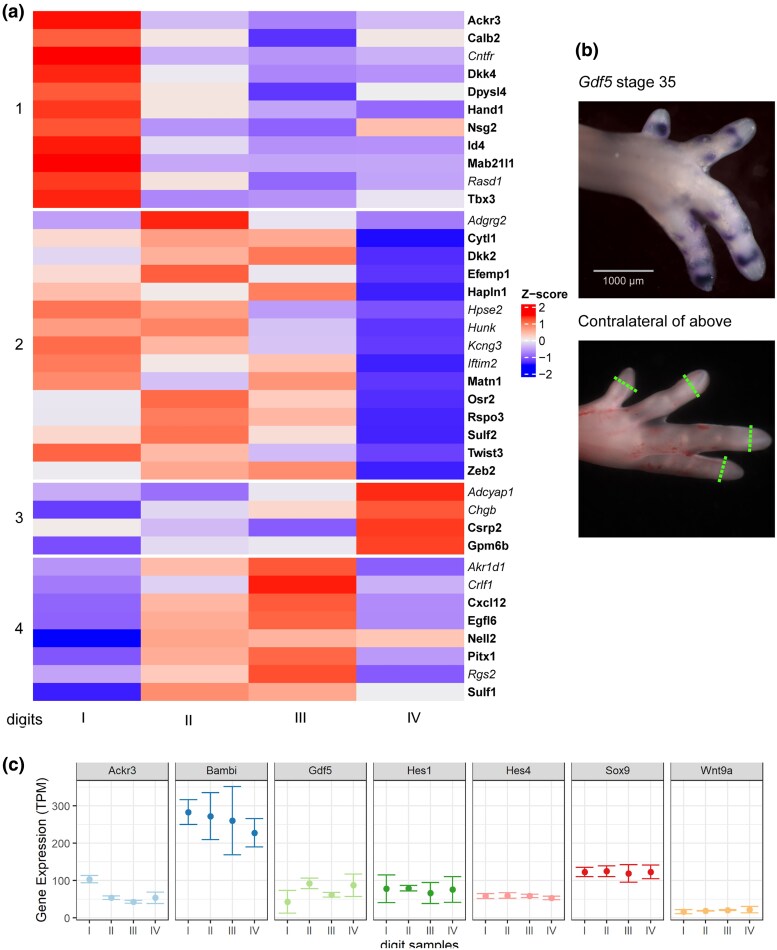
Candidate cyclical genes revealed by bulk transcriptomic analysis of stage 35 chicken toe tip tissues. a) Heatmap of 38 genes with differential expression >25 transcripts per million. Genes in bold type are genes known to be involved in limb development. b) Chicken embryo hindlimbs (stage 35) indicating the source of tissues for bulk transcriptomics. Upper figure: in situ for *Gdf5* showing lack of expression in the PFR of digits I–III and expression in the PFR of digit IV. Lower picture: the contralateral hindlimb of the same embryo with a green dotted line indicating the excision plane. c) Profile plots of selected genes. Note the difference in transcript abundance for *Ackr3* and *Gdf5* between the four digits suggesting that they are at different phases of the cycle at this moment. *Hes1* and *Hes4* appear not to be cyclical in any of our analyses including in situ hybridization (supplementary fig. S2, Supplementary Material online). The expression of *Wnt9a* at this stage has an equally low expression across all four digits, but in a time series of limbs, it can be seen to be cyclical in expression (Fig. 6).

**Fig. 6.**
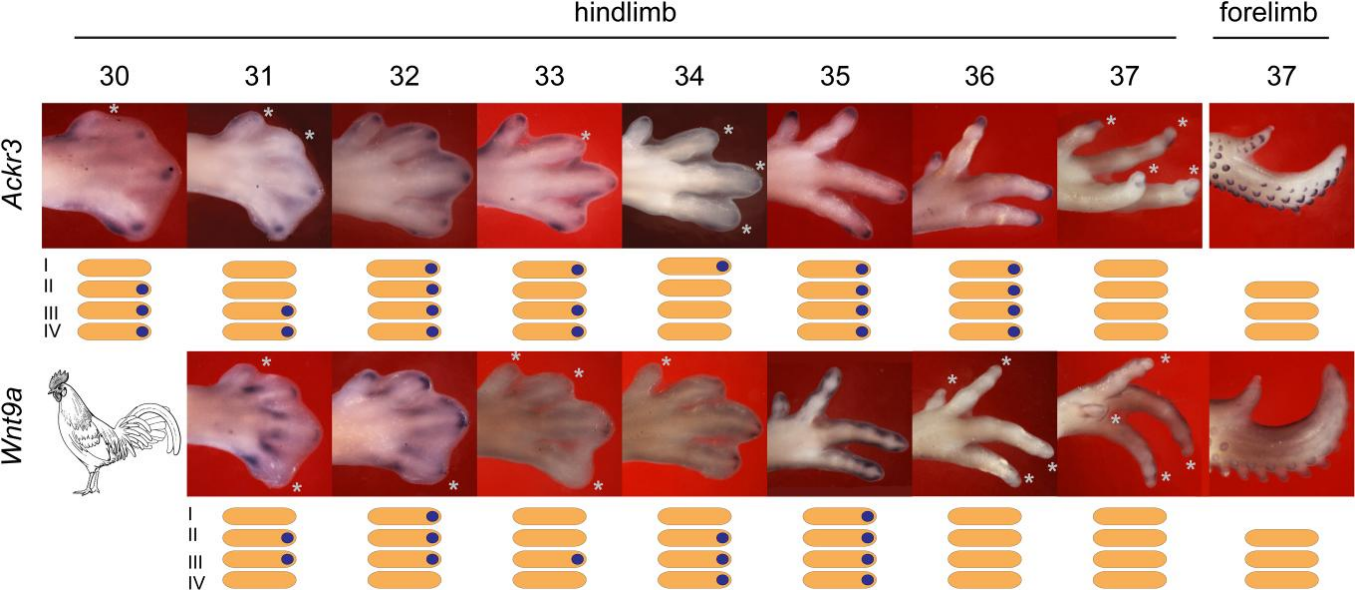
*Ack3* and *Wnt9a* show cyclical expression in the chicken embryo hindlimb PFRs. Anterior is to the top, distal to the right. Asterisks denote PFRs in which there is no detectable expression. Below the photographs is a schematic representation of the expression of the genes. As can be seen by comparing different digits at different stages, the expression of *Ackr3* and *Wnt9a* is cyclical. For example, *Ackr3* is “on” (expressed) in digit II at stage 30, “off” (not expressed) at stage 31, on at stages 32 and 33, off at stage 34, on at stages 35 and 36, and off at stage 37, after the phalanx-to-claw transition. An example of the cyclical expression of *Wnt9a* is provided by digit I; it is off at stage 31, on at stage 32, off at stages 33 and 34, on at stage 35, and off at stages 36 and 37. The lack of expression of these genes at later stages is not due to failure of the in situ protocol, because a stage 37 forelimb from the same embryos (far right column) shows expression of *Ackr3* and *Wnt9a* (fainter) in the feather buds.

To identify genes expressed cyclically during phalanx formation, we carried out transcriptome sequencing on the microdissected tips of chicken hindlimb digits (stage 35, three biological replicates per digit; see Fig. 5). We processed the contralateral limbs of the same embryos for wholemount in situ hybridization with a *Gdf5* probe to determine the oscillation phase of each digit tip at the time of tissue harvesting (Fig. 5). We then carried out differential gene expression analysis between these digit tip transcriptomes to detect putative oscillation genes whose expression was out of phase in the different digit tips (Fig. 5; supplementary fig. S4, Supplementary Material online). Applying a threshold level of 25 transcripts per million yielded 38 candidate cyclical genes (Fig. 5) which were screened against the literature and public databases to refine the list. This left 16 genes which we supplemented with other candidates from the literature (including *Hes1* and *Hes4*). In situ hybridization on all 16 candidates identified *Ackr3* and *Wnt9a* as novel oscillation genes expressed in the PFR (Fig. 6).

*Ackr3* is a chemokine receptor involved in bone differentiation (Liu et al. 2023). It is also expressed during limb development (Kee and Bronner-Fraser 2001; Tokuzawa et al. 2010; Duffield et al. 2021) and is a regulator of circadian variation in glucocorticoid levels (Quinn et al. 2018). The cyclical expression of *Ackr3* that we detected is shown in Fig. 6. *Wnt9a* is a member of the Wnt family of secreted signaling proteins that play important roles in limb development and many other biological processes (Nusse and Clevers 2017). We find *Wnt9a* to be cyclically expressed in the PFR (Fig. 6). Interestingly, we did not find cyclical expression of *Hes1* and *Hes4* (supplementary fig. S2, Supplementary Material online), even though these two genes are associated with the somite clock (Maia-Fernandes et al. 2024) and the early limb development clock (Jouve et al. 2000; Vasiliauskas et al. 2003; Sheeba et al. 2012, 2016).

### Synchronous Changes in Developmental Gene Expression at the Transition Between Phalanx Formation and Claw Development

We find that a group of developmental patterning genes are downregulated synchronously when phalanx formation ends and claw development begins around stage 36. These downregulated genes include *Sox9*, *Gdf5*, *Wnt9a*, *Ackr3*, *Hes1*, *Hes4*, *Id4*, and *Bmpr1b* (supplementary fig. S2, Supplementary Material online). The expression of the PFR marker, *Sox9*, terminates at stage 36 in all claw-bearing digits in all species studied (Fig. 2; supplementary fig. S5, Supplementary Material online). At the same stage that phalanx formation terminates (stage 36), a group of genes associated with claw development is upregulated in all claw-bearing digits in all species studied. These genes include *Bambi*, *Msx1*, and *Msx2* (Fig. 2; supplementary figs. S3 and S6, Supplementary Material online).

These data pinpoint a developmental window of phalanx pattering, in all species studied, from stage 29 to 36, a finding that is further supported by our analysis of *Gdf5* expression patterns in the developing interphalangeal joints (Fig. 3b and c). The exceptions among the digits studied were the clawless digits in the bird wing and the posterior digits of the turtle and crocodilians (Crocodilia). Thus, at stage 34, the clawless chicken wing digit IV shows downregulation of *Sox9*. This digit is reduced in all birds and shows apoptosis in the tissue at its apex (Fig. 4 in de Bakker et al. 2021). In the turtle and crocodilians studied here, *Sox9* expression continues in the PFR as late as stage 37, a stage at which it has already terminated in the clawed digits of the same limb (Fig. 2; supplementary fig. S5, Supplementary Material online).

## Discussion

We identify a conserved developmental time window for phalanx formation, in all the amniotes we studied, corresponding to chicken stages 28–36 (Fig. 3; supplementary figs. S1 and S2, Supplementary Material online). We also identify two novel cyclically expressed genes, *Ackr3* and *Wnt9a*, in the chicken PFR (Fig. 6). We show that the frequency of cyclical expression of *Gdf5* in the PFR is correlated with the number of phalanges developed in that digit. Finally, the PFR in clawless digits remains active after it has disappeared in clawed digits, as indicated by continued *Sox9* expression (Fig. 2). Together, these observations suggest that shifts in the timing of gene expression may be responsible for significant evolutionary changes in the phenotype of the amniote autopod. Such changes in developmental timing are known as transcriptional or molecular heterochrony (Zákány et al. 1997; Kim et al. 2000; Skaer et al. 2002; Bickelmann et al. 2012). We used *Gdf5*, *Sox9*, *Bambi*, and other genes as “reporters” of this heterochrony. We do not suggest that these genes are the most proximal (causal) members of the relevant regulatory pathway, however.

In all 13 species studied here, the phalanx–claw transition takes place simultaneously at stage 36 in all digits (Fig. 2). At this stage, the digits are developing autonomously from one another (there is no tissue web connecting them). This supports the hypothesis that each PFR of each digit has its own “developmental clock settings”. Our species sample encompasses mammals, squamates, testudines, crocodilians, and birds (Fig. 1). Furthermore, the synchronous downregulation of *Sox9* expression in the digits can be seen in a published study of the Iberian mole, *Talpa occidentalis*, and the North American least shrew, *Cryptotis parva* (Fig. 2 in Mitgutsch et al. 2012).

In the chicken and many other sauropsids, each digit develops a different total number of phalanges, but in the same time window, ending at stage 36. At this stage, the phalanx–claw transition occurs in the same stage range (chick stages 36 and 37) in all species studied (Fig. 2). We suggest that the frequency is set for each digit before stage 29, i.e. during early limb patterning when the zone of polarizing activity and AER are still active (Summerbell 1974; Riddle et al. 1993; Galloway and Tabin 2008; Pickering et al. 2018). Therefore, the PFR of each digit has its own internal clock that runs autonomously at a digit-specific speed, and this clock runs in a fixed time window between stages 29 and 36 (Fig. 3b and c).

In the PFR, at the moment of the phalanx–claw transition (chicken stage 36), there are two notable transcriptional changes. First, numerous developmental patterning genes, including the PFR marker *Sox9*, show downregulation; and second, *Bambi*, *Msx1*, and *Msx2*, which are markers of claw development, show upregulation (supplementary figs. S3 and S6, Supplementary Material online) and also seen in the turtle (Cordeiro et al. 2020). Importantly, we find that this transition is delayed beyond chicken stage 36 in digits that lack claws. Thus, in the Chinese soft-shelled turtle, and in the crocodilians studied here, *Sox9* expression continues after chicken stage 37 in the posterior clawless digits (Fig. 4; supplementary fig. S5, Supplementary Material online). In digits I, II, and III, *Bambi* is still expressed after the phalanx-transition. The claw-related gene *Bambi* is never expressed in the clawless posterior digits IV and V.

Another important fact is that clawless digits in amniotes often show hyperphalangy. For example, the Chinese soft-shelled turtle has a variable number of phalanges in its clawless (posterior) digits, and this number sometimes exceeding the ancestral number (Delfino et al. 2010). In that paper, the authors suggested that hyperphalangy in digits IV and V of *Pelodiscus sinensis* might be due to prolonged growth of those two digits. Also the American alligator develops four phalanges in fore limb digit V instead of the ancestral three (Müller and Alberch 1990). As noted above, both *Pelodiscus sinensis* and the crocodilians do not express the claw development marker *Bambi*—but still express *Sox9*, the PFR marker, in digits IV and V at this transitional stage (Fig. 2). These clawless digits fail to undergo the transition from phalanx formation to claw development. This failure could represent the lifting of a developmental constraint, allowing the digit to progress to a state of hyperphalangy. Several other species also show a combination of claw loss and hyperphalangy. The *Tarentola* geckos (Gekkonidae) have lost a claw in digit I and have hyperphalangy: three instead of the ancestral two phalanges (Haacke 1976; Carranza et al. 2002; Khannoon et al. 2024). The New World fossil turtles (Pan-Trionychidae) show loss of claws combined with hyperphalangy in digits IV and V (Vitek and Joyce 2015) as do fossil mosasaurs and ichthyosaurs in all its digits (Nopcsa 1903; Fedak and Hall 2004; Thewissen et al. 2007).

These considerations suggest two developmental mechanisms underlying the evolution of phalanx number. The first is seen in clawed amniote digits. In these digits, we know of no case where the ancestral number of phalanges is exceeded. By contrast, phalanx number can decrease if the PFR “oscillation” runs more slowly. This, we suggest, is what has happened in the clawless wing digits II–IV of the zebra finch and the clawed digits III and IV of the mouse forelimb (Fig. 1). The second mode for evolutionary changes in phalanx number is seen in digits without a claw. In these digits, the constraint that imposes a maximum limit on the number of phalanges has gone. Therefore, it is possible for hyperphalangy to develop. The Chinese soft-shelled turtle shows both processes; in the clawed digit III, one phalanx is lost and the clawless digits IV and V have a variable number of phalanges (3–6 and 2–5, respectively; Delfino et al. 2010).

The flippers of cetaceans (whales and dolphins) also show hyperphalangy (Richardson and Oelschlager 2002). The digits of these flippers have no claws (Flower 1870; Cooper and Dawson 2009). Not only does the cetacean flipper show hyperphalangy but, as in the Chinese soft-shelled turtle digits IV and V, the number of phalanges shows intraspecific variation (Quiring and Harlan 1953; Delfino et al. 2010). In support of our model, it has recently been shown that there is relaxed selection on genes associated with flipper development in cetaceans (Telizhenko et al. 2024). It could be argued that a loss of claws combined with hyperphalangy is purely a function of selection for a flipper, and not a consequence of developmental constrains as our model suggests. However, the flipper of the manatee (*Trichechus* sp.), an aquatic species, has claws and no hyperphalangy (Quiring and Harlan 1953). Clawless does not always lead to hyperphalangy; for example, most bird wings have lost both claws and phalanges (Fig. 1). Thus, the zebra finch wing has no claws and has just four of the ancestral 17 phalanges (Fig. 1).

In summary, our findings suggest that there are at least two major control points in amniote digit development: (i) the variable oscillation frequency of phalanx formation and (ii) the conserved developmental stage of the phalanx–claw transition. The variable oscillation frequency can explain the loss of phalanges during evolution, whereas the loss of the phalanx–claw transition unlocks a developmental constraint on maximal phalanx number. We call these “control points” because they appear to be open to natural selection for limb phenotype, as illustrated by the various limb adaptations seen in our species sample. Furthermore, our findings suggest a model in which even highly conserved genetic programs nonetheless contain developmental “hotspots” such as those postulated in *Drosophila* (Richardson and Brakefield 2003). These hotspots are subject to evolutionary tinkering (Richardson and Brakefield 2003; Duboule et al. 2007; Brakefield 2011), which can lead to adaptive changes in phenotype.

We believe that our model of phalanx and claw formation may lead to new insights into digit evolution and development. Evolutionary differences in phalanx number, and evolutionary loss of claws, can be simply explained in terms of evolutionary “tinkering” (Duboule et al. 2007; Brakefield 2011) with the intrinsic digital clock (Pascoal et al. 2007; Chinnaiya et al. 2014; Saiz-Lopez et al. 2017). The development of phalanges and claws is normally coupled by a developmental constraint which, when lifted, allows more phalanges to develop (hyperphalangy). In principle, our findings may help us to understand the adaptive radiation of amniotes in terms of phenotypic character evolution, and the evolution of developmental mechanisms.

## Materials and Methods

### Collection of Embryos

In total, 467 embryos of 13 amniote species were processed and analyzed (supplementary table S1 and S2, Supplementary Material). The house mouse (*Mus musculus*) embryos were bred and harvested in compliance with the Netherlands Law on Animal Testing (*Wet op de Dierproeven*), licence number 14,167u, at Leiden University. Sauropsid embryos were harvested in compliance with guidelines and regulations concerning the use of experimental animals, namely European Union (EU) directive no. 2010/63/EU and its implementation in The Netherlands, the *Wet op de Dierproeven*. The embryos used in these experiments were too young to have reached the stage of exogenous (heterotrophic) feeding where the yolk sac has been exhausted (this takes place at hatching). Therefore, the experiments reported here are not considered as animal experiments under the EU directive and Dutch law mentioned above.

The chicken (*Gallus gallus*), duck (*Anas platyrhynchos*), ostrich (*Struthio camelus*), and emu (*Dromaius novaehollandiae*) eggs were from commercial breeders. Some additional emu embryos were provided by Dr. John Young of the Clifford Tabin Lab, Department of Genetics, Harvard Medical School, United States of America. The zebra finch (*Taeniopygia guttata*) eggs were a gift from Prof. Dr. Carel J. ten Cate and Dr. Katharina Riebel, both from the Animal Sciences Cluster, Institute of Biology Leiden, Leiden University, the Netherlands. The eggs of the broad-snouted caiman (*Caiman latirostris*) were a gift from René Hedegaard, Director of Krokodille Zoo, Eskilstrup, Denmark. The Nile crocodile (*Crocodylus niloticus*) eggs are from La Ferme Aux Crocodiles, Pierrelatte, France, and the dwarf crocodile (*Osteolaemus tetraspis*) eggs were a gift from Walter Getreuer, Serpo, Rijswijk, the Netherlands, under Convention on International Trade in Endangered Species of Wild Fauna and Flora (CITES) certificate 21NL194589/20 from the Ministry of Argiculture, Nature and Food Quality, CITES Management Authority, The Hague, The Netherlands. Chinese soft-shelled turtle (*Pelodiscus sinensis*) embryos were collected by Dr. Tatsuya Hirasawa, Evolutionary Morphology Laboratory, RIKEN, Japan. Most of the Central bearded dragon (*Pogona vitticeps*) eggs were purchased from commercial breeders, except for one clutch which was donated by Bregeta Demmer and Jordy Hol, Purmerend, the Netherlands.

The tokay gecko (*Gekko gecko*) embryos were collected by Luthfi Nurhidayat. Thirty-nine adult tokay geckoes were captured in the vicinity of Yogyakarta, Indonesia, under a license issued by the Ministry of Environment and Forestry, the Republic of Indonesia (permit number: SK.83/KSDAE/SET/KSA.2/5/2021 signed on 2021 May 7), and a recommendation letter issued by The Indonesian Institute of Sciences (Lembaga Ilmu Pengetahuan Indonesia/LIPI; Recommendation Number: B-2158/IV/KS.01.04/3/2021 signed on 2021 March 19). All experimental and surgical procedures needed for samples collection were done at Faculty of Biology, Universitas Gadjah Mada, Yogyakarta, Indonesia, with the approval from The Ethical Committee of Integrated Laboratory for Research and Testing (Laboratorium Penelitian dan Pengujian Terpadu/LPPT) Universitas Gadjah Mada (Ethical Clearance number: Ref. 00014/04/LPPT/IV/2021 signed on 2021 April 30). The samples were transported from Universitas Gadjah Mada, Yogyakarta, Indonesia, to Institute of Biology Leiden, the Netherlands, under transport permission from the Ministry of Environment and Forestry, the Republic of Indonesia (No. 05717/IV/SATS-LN/2021 signed on June 2, 2021).

All embryos, unless stated otherwise above, were incubated and harvested at the Institute of Biology Leiden (IBL), Leiden University, the Netherlands. After candling the eggs (in the case of the sauropsids studied), we removed the embryos into ice-cold phosphate buffered saline (PBS) in a Petri dish. The amnion was removed, and the embryo fixed in ice-cold 4% formaldehyde in PBS at 4 °C overnight. The next day, they were dehydrated in a graded methanol series and stored in 100% methanol at −20 °C. Staging for all species was based on the chicken stages in Hamburger and Hamilton for easier comparison (Hamburger and Hamilton 1951). Hatching times vary greatly from 14 days in the zebra finch to around 80 days in the crocodiles (supplementary table S3, Supplementary Material online). During development we estimate the phalanx-forming period between 2.25 days of incubation in the zebra finch to 15 days in the crocodilians and the bearded dragon (supplementary table S3, Supplementary Material online).

### Whole Mount In Situ Hybridization

#### In Situ Hybridization Probes

All probes were made in-house and their sequences deposited at The National Center for Biotechnology Information (NCBI) (supplementary tables S4 and S5, Supplementary Material online). The *Bambi* and *Gdf5* gene expression on all bird embryos were done with a chicken probe and for *Sox9* with an emu probe. The chicken *Hes1* and *Hes4* (Hes Family BHLH Transcription Factor) coding sequences start with 300 almost identical base pairs. To be confident, they did not cross-hybridize, we amplified with forward primers after these identical sequences and 3′ RACE primers (**R**apid **A**mplification of **c**DNA ends; Scotto-Lavino et al. 2006). The resulting probe templates also include the 3′ untranslated region. All crocodilian in situ experiments were done with Nile crocodile probes. For synthesizing the probes, we isolated total RNA from an embryo using Trizol (Invitrogen) and carried out reverse transcription using SuperScript III (Invitrogen). Polymerase chain reaction (PCR) was performed on these templates using specific primers, and the PCR products were cloned in the TOPOTA-PCRII vector (Invitrogen). The inserted amplicons were checked by PCR with M13-pUC primers located on the TOPOTA-PCRII plasmid and checked on an agarose gel. When they were of the right length, they were Sanger sequenced. After confirming the sequence results by Basic Local Alignment Search Tool (BLAST) searching, the positive results were used as templates for making the digoxigenin labeled antisense RNA probes.

#### Hybridization Protocol

The embryos were rehydrated through a graded methanol series, lightly digested with proteinase K (20–40 µg/ml in PBS) for 20 min and postfixed in 4% formaldehyde in PBS after several washes in PBST (PBS pH 7.2 with 0.1% Tween-20). This was followed by a prehybridization step at 60 °C for at least 3 h or until the embryo had sunk. The hybridization mixture consisted of 50% formamide, 2% Boehringer blocking powder, 5× SSC (dilluted form 20× standard sodium citrate buffer, 3 M sodium chloride, 0.3 M sodium citrate, pH 7), 1 mg/ml total RNA, 50 μg/ml heparin, 0.1% Triton X-100, 0.1% CHAPS detergent (3-[(3-cholamidopropyl) dimethylammonio]-1-propanesulfonate) and 5 mM EDTA. After the prehybridization mix was removed, we added 400 ng/ml specific probe to fresh hybridization mixture preheated to 60 °C before adding it to the embryo. The embryos were incubated in this mix at 60 °C overnight with slow shaking. The next day, the specific probe mixture was removed, collected, and stored at −20 °C for reuse.

Several stringent washes were done at 60 °C to remove nonspecifically bound probe [2× SSC, 0.1% CHAPS, 50% formamide], [2× SSC 0.1% CHAPS], and [0.2× SSC, 0.1% CHAPS]. After washing several times at room temperature with TBST (0,1 M Tris Buffered Saline, 0.1% Tween-20, pH 7.5), the embryos were preincubated with heat inactivated 10% sheep serum in TBST for 90 min at room temperature followed by overnight incubation with sheep antidigoxigenin conjugated to alkaline phosphatase (Roche; 1:5,000 dilution in 10% sheep serum in TBST at 4 °C overnight). Next day, the nonspecifically bound antibodies were washed away by several washes with TBST of which one was overnight. The embryos were brought to a higher pH by washing them in NTT buffer (**N**aCl, **T**ris/HCL, **T**ween: 0.1 M sodium chloride, 0.1 M Tris/HCl, 0.1% Tween-20, pH 9.5). The enzyme reaction of alkaline phosphate with BM purple (Roche 11442074001) as substrate results in a blue precipitate. The development of the stain was checked regularly and stopped by washing several times in TBST, removing the substrate and chromogens, and lowering the pH.

### Analysis of *Gdf5* Expression

To count the number of phalanges formed during development, we used the expression patterns of *Gdf5* as a proxy. *Gdf5* is first expressed in the PFR and persists during interphalangeal joint differentiation (Storm and Kingsley 1999; Suzuki et al. 2008; Huang et al. 2016). The scoring of the phalanges as tabulated in Fig. 3b and c is based on the unstained tissue between, and distal to, the *Gdf5* expression domains that indicate the joint (Figs. 3 and 4).

The *Gdf5* expression intensity (the purple stained joints in each digit) was measured in order to compare the patterns quantitatively. Digital photographs of 18 bearded dragon limbs shown in Fig. 4a were imported into the image analysis program FIJI (Fiji is just ImageJ; Schneider et al. 2012). In FIJI, we used a custom-made line analysis tool (written by J.W.) to obtain the intensity profiles of all digits. This was done by manually drawing a line (see the yellow line in Fig. 4b as an example) of which the intensity of every pixel (minimal 184 and maximum 1,195) was measured in Fiji from distal to proximal. For each autopod, the distance (number of pixels) was mathematically “stretched” to normalize the lengths of the digits in each autopod. This normalization was necessary because the five digits of each autopod differ in length, and because pixels are squares and their width is shorter than their diagonal. The stain intensities along this line were plotted against the normalized distance in a scatter plot giving a pseudo-line rendering in the final graphs (Fig. 4b, the green line which are in fact a row of dots). The correlations between the resulting digit profiles in each individual autopod were calculated using the Microsoft Excel “CORREL” function. These correlations were averaged over all 18 fore- and hindlimbs, see the developmental series in Fig. 4a.

### Differential Gene Expression Analysis With Bulk Transcriptomics

Using stage 35 chicken embryos, one hindlimb was used for sampling tissue for RNA extraction (dissected, snap frozen in liquid nitrogen, and stored at −80 °C) while the contralateral hindlimb of the same embryo was fixed for *Gdf5* in situ hybridization. On the base of the *Gdf5* results, we selected three limbs with no expression in digits I–III in the tips but with *Gdf5* expression in the tip of digit IV to extract RNA for transcriptome sequencing (Fig. 5). The RNA of the toe tips was isolated with ReliaPrep™ RNA Tissue Miniprep System (Promega) with minor modifications and sent to GenomeScan (Leiden, the Netherlands) on dry ice for transcriptome sequencing.

The library preparation and RNA sequencing were done by GenomeScan with in-house protocols. The sequencing platform used was Illumina NovaSeq 600 with a read length of 2 × 150 bp. Transcriptome data analysis was carried out within the High Performance Computing Facility, Leiden University (ALICE). The transcripts of all samples were quantified from the sequencing data using Salmon 1.6.0 (https://salmon.readthedocs.io/en/latest/salmon.html; Patro et al. 2017) with tanuki pipeline (https://github.com/RxLoutre/tanuki.git). The transcript quantification used the chicken genome as a reference (https://www.ncbi.nlm.nih.gov/datasets/genome/GCF_016699485.2/). The sequence data quality check with FastQC (https://github.com/s-andrews/FastQC) was included in the pipeline.

The quantification files were then analyzed with 3DRNAseq pipeline (https://github.com/wyguo/ThreeDRNAseq; Guo et al. 2021) utilizing limma-voom weights pipeline of limma R package for differential gene expression analysis (Ritchie et al. 2015). We compared each individual digit to all other digits as contrast groups. The log_2_ fold change (L_2_FC) of gene/transcript abundance was calculated based on contrast groups, and significance of expression changes was determined using a *t*-test. *P*-values of multiple tests were adjusted with the Benjamini–Hochberg test to correct the false discovery rate (Benjamini and Yekutieli 2001). A gene/transcript was considered significantly differentially expressed in a contrast group if it had an adjusted *P*-value < 0.05 and L_2_FC ≥ 1. The results were visualized using the ComplexHeatmap (Gu et al. 2016) package and ggplot2 on Rstudio, with the cut-off at 25 transcripts per million.

## Supplementary Material

msaf113_Supplementary_Data

## Data Availability

See supplementary tables S4 and S5, Supplementary Material online, for the probe sequences accession numbers. All raw RNA sequence data used for transcriptome analysis have been deposited on NCBI under BioProject number PRJNA1141260 and are publicly available as of the date of publication. The source code for the line analyses of clock rate in joint development can be found at https://sites.imagej.net/Willemsejj/. All other data that support the findings of this study are available from the corresponding author upon reasonable request.
