# Supplementary material for: Changes in Evolutionary Developmental Control Points in the Amniote Limb May Explain Hyperphalangy
Source: Mol Biol Evol. 2025 Jun 9;42(6):msaf113. doi: 10.1093/molbev/msaf113 (PMC12164292; doi:10.1093/molbev/msaf113)
Supplement: msaf113_Supplementary_Data [file msaf113_supplementary_data.zip › Supplementary Tables and Figures.docx]

# Supplementary Tables

Supplementary_Table 1. **List of species studied**. Nomenclature according to Pubmed Taxonomy (www.ncbi.nlm.nih.gov/Taxonomy/Browser/).

| **Scientific name** | **Common name** | **Taxa** |
| --- | --- | --- |
| *Mus musculus* | House mouse | Mammalia: Rodentia |
| *Pogona vitticeps* | Central bearded dragon | Squamata: Agamidae |
| *Gekko gecko* | Tokay gecko | Squamata: Gekkonidae |
| *Pelodiscus sinensis* | Chinese soft-shelled turtle | Testudines; Trionychidae |
| *Caiman latirostris* | Broad-snouted caiman | Crocodylia; Alligatoridae |
| *Osteolaemus tetraspis* | Dwarf crocodile | Crocodylia; Crocodylidae |
| *Crocodylus niloticus* | Nile crocodile | Crocodylia; Crocodylidae |
| *Struthio camelus* | African ostrich | Aves: Palaeognathae |
| *Dromaius novaehollandiae* | Emu | Aves: Palaeognathae |
| *Rhea americana* | Greater rhea | Aves: Palaeognathae |
| *Gallus gallus* | Chicken | Aves: Neognathae: Galloanserae |
| *Anas platyrhynchos* | Duck | Aves: Neognathae: Galloanserae |
| *Taeniopygia guttata* | Zebra finch | Aves: Neognathae: Passeriformes |

Supplementary_Table 2 **Number of *in situ* hybridisations and embryos used**. One in situ hybridisation is a pair of forelimbs and hindlimbs, left or right, from the same embryo. Contralateral limbs of the same individual have sometimes been used to visualise the expression patterns of two different genes, therefore the total of embryos is less than the total no of in situ hybridisations. Genes we hoped to see oscillating in the chicken are listed in Supplementary_Figure 5.

| Scientific name | *Bambi* | *Sox9* | *Gdf5* | *In situ* hybridisations | embryos |
| --- | --- | --- | --- | --- | --- |
| *Mus musculus* | 4 |  | 4 | 8 | 4 |
| *Pogona vitticeps* | 25 | 9 | 25 | 59 | 34 |
| *Gekko gecko* | 2 | 2 | 3 | 7 | 5 |
| *Pelodiscus sinensis* | 5 | 9 | 12 | 26 | 24 |
| *Caiman latirostris* | 4 | 0 | 3 | 7 | 5 |
| *Osteolaemus tetraspis* | 3 | 2 | 2 | 7 | 5 |
| *Crocodylus niloticus* | 10 | 6 | 5 | 21 | 16 |
| *Struthio camelus* | 7 | 4 | 4 | 15 | 11 |
| *Dromaius novaehollandiae* | 4 | 9 | 1 | 13 | 12 |
| *Rhea americana* | 3 | 0 | 0 | 3 | 3 |
| *Gallus gallus* | 30 | 16 | 18 | 64 | 54 |
| *Anas platyrhynchos* | 20 | 17 | 4 | 41 | 35 |
| *Taeniopygia guttata* | 27 | 13 | 14 | 54 | 45 |
| Total | **140** | **87** | **91** | **318** | **253** |

Supplementary_Table 3. **Hatching times and estimates of the corresponding incubation days at which phalanx forming takes place** (i.e. the stages equivalent to HH stages 28–36 HH). The estimation of the duration of phalanx formation is variable as is the incubation time in most animals studied. At chicken stage 28 the limb is formed as a paddle and at stage 36 the first indications of the claw are present. At stage 26 the first *Sox9* expression is seen in the developing digits and in stage 36 the last *Sox9* expression is seen in the phalanx forming region in the digits tips. In all studied animals the digit pattern is assembled during this developmental period (de Bakker, Fowler et al. 2013, Montero, Lorda-Diez et al. 2017, de Bakker, van der Vos et al. 2021). All the species we studied hatch at different times from 14 days in zebra finches to more than 100 days in the Nile crocodile, see above table. From the species in the above table only the zebra finch is precocial but the extant of development times in the studied species is still significant different. The developmental phenotypes of the limbs however are comparable (figures 2 and 3, sup. Figures 1, 2 and 3) as are the expression patterns we detected. Also, with exception of the zebra finch, the hatching times and phalanx development times for each studied species are variable. Seen this variation in hatching time within and between species a general time estimate for the development of each phalanx will be always be very imprecise.

| **Scientific name** | **Common name** | **Hatching time (days)** | **phalanx forming period (days)** | **Reference** |
| --- | --- | --- | --- | --- |
| *Taeniopygia guttata* | zebra finch | 14 | 2.25 | (Murray, Varian-Ramos et al. 2013) |
| *Gallus gallus* | chicken | 20–22 | 4–5 | (Hamburger and Hamilton 1951) |
| *Dromaius novaehollandiae* | emu | 50–56 | 11–12 | (Nagai, Mak et al. 2011)  Estimate of their figure 2 |
| *Struthio camelus* | ostrich | 39–42 | 7–8 | (Gefen and Ar 2001) |
| *Caiman latirostris* | broad-snouted caiman | 70–85 | 13–17 | (Iungman, Pina and Siroski 2008) |
| *Crocodylus niloticus* | Nile crocodile | 74–103 | 9–11 | (Peterka, Sire et al. 2010) |
| *Pelodiscus sinensis* | Chinese soft-shelled turtle | 45–50 | 14–16 | (Tokita and Kuratani 2001) |
| *Pogona vitticeps* | Central bearded dragon | 59–67 | 15–20 | (Melville, Hunjan et al. 2016, Whiteley, Holleley et al. 2017) |

Supplementary_Table 4 **NCBI** **Accession numbers of probes used.** We have deposited the sequences in the NCBI (National Center for Biotechnology Information) database.

| Scientific name | *Bambi* | *Sox9* | *Gdf5* |
| --- | --- | --- | --- |
| *Mus musculus* | KX896990 |  | KX688160 |
| *Pogona vitticeps* | KX896989 | MF185833 | KX688159 |
| *Gekko gecko* | OK338007 | OK338009 | OK338008 |
| *Pelodiscus sinensis* | KX896991 | MF185834 | KX688158 |
| *Crocodylus niloticus* | KX896988 | JQ717196 | KX688157 |
| *Gallus gallus* | KX896988 |  | KX688154 |
| *Dromaius novaehollandiae* |  | JQ717195 |  |

Supplementary_Table 5 **Probes used to detect possible oscillating genes in the chicken hindlimb** One *in situ* hybridisation is staining one pair of fore- and hind limbs, left or right. Contralateral limbs of the same individual have been used to visualise expression patterns of two different genes, therefore the total of embryos is less than the total of expression studies.

| **Gene** | **Accession number**  **Found by BLAST** | **Number of *in situ* hybridisation experiments** | **Expression** |
| --- | --- | --- | --- |
| ACKR3 (CXCR7) | NM_001083362.2 | 21 | **PFR oscillating** (Figure 6) |
| BMP4 | NM_205237.4 | 2 | Interdigital |
| BMPR1B | NM_205237.4 | 10 | PFR |
| DKK2 | XM_015276579.4 | 3 | Not in PFR |
| FGF8 | U55189.1 | 11 | PFR not oscillating |
| FGF10 | NM_204696.2 | 9 | PFR not oscillating |
| GLI1 | U60762.1 | 2 | Not in PFR |
| HAND1 | NM_204965.2 | 4 | Entire digit margin |
| HES1 | XM_040679737.2 | 27 | PFR not oscillating |
| HES4 | NM_001005848.3 | 28 | PFR |
| ID4 | NM_001396104 | 9 | PFR |
| MSX1 | KX897001 | 11 | Entire digit margin shift to PFR at stage 36 |
| MSX2 | NM_204559.2 | 2 | Entire digit margin shift to PFR at stage 36 |
| PITX1 | NM_001167686.2 | 3 | Interphalangeal joints |
| RELN | NM_001305123.2 | 14 | 29 AER 30-34 distal in III and IV 35-36 PFR |
| TFAP2B | NM_204895.2 | 11 | Interdigital, weak |
| TBX3 | NM_001270878.2 | 5 | Interdigital |
| SULF2 | XM_040688668.2 | 4 | Interphalangeal joints |
| WNT5A | KX897002 | 9 | Entire digit margin |
| WNT9A | NM_204981.3 | 25 | **PFR oscillating** Figure 6) |
| ZEB2 | NM_001318466.2 | 3 | Interdigital |
| **Total** |  | **214** |  |
