# Supplementary material for: Changes in Evolutionary Developmental Control Points in the Amniote Limb May Explain Hyperphalangy
Source: Mol Biol Evol. 2025 Jun 9;42(6):msaf113. doi: 10.1093/molbev/msaf113 (PMC12164292; doi:10.1093/molbev/msaf113)
Supplement: msaf113_Supplementary_Data [file msaf113_supplementary_data.zip › Supplementary Tables Figures and information.pdf]

Supplementary Tables

Supplementary_Table 1. **List of species studied**. Nomenclature according to Pubmed Taxonomy (www.ncbi.nlm.nih.gov/Taxonomy/Browser/).

**Scientific name**	**Common name**	**Taxa**
Mus musculus	House mouse	Mammalia: Rodentia
Pogona vitticeps	Central bearded dragon	Squamata: Agamidae
Gekko gecko	Tokay gecko	Squamata: Gekkonidae
Pelodiscus sinensis	Chinese soft-shelled turtle	Testudines; Trionychidae
Caiman latirostris	Broad-snouted caiman	Crocodylia; Alligatoridae
Osteolaemus tetraspis	Dwarf crocodile	Crocodylia; Crocodylidae
Crocodylus niloticus	Nile crocodile	Crocodylia; Crocodylidae
Struthio camelus	African ostrich	Aves: Palaeognathae
Dromaius novaehollandiae	Emu	Aves: Palaeognathae
Rhea americana	Greater rhea	Aves: Palaeognathae
Gallus gallus	Chicken	Aves: Neognathae: Galloanserae
Anas platyrhynchos	Duck	Aves: Neognathae: Galloanserae
Taeniopygia guttata	Zebra finch	Aves: Neognathae: Passeriformes

Supplementary_Table 2 **Number of *in situ* hybridisations and embryos used**. One in situ hybridisation is a pair of forelimbs and hindlimbs, left or right, from the same embryo. Contralateral limbs of the same individual have sometimes been used to visualise the expression patterns of two different genes, therefore the total of embryos is less than the total no of in situ hybridisations. Genes we hoped to see oscillating in the chicken are listed in Supplementary_Figure 5.

**Scientific name**	***Bambi***	***Sox9***	***Gdf5***	***In situ* hybridisations**	**embryos**
Mus musculus	4		4	8	4
Pogona vitticeps	25	9	25	59	34
Gekko gecko	2	2	3	7	5
Pelodiscus sinensis	5	9	12	26	24
Caiman latirostris	4	0	3	7	5
Osteolaemus tetraspis	3	2	2	7	5
Crocodylus niloticus	10	6	5	21	16
Struthio camelus	7	4	4	15	11
Dromaius novaehollandiae	4	9	1	13	12
Rhea americana	3	0	0	3	3
Gallus gallus	30	16	18	64	54
Anas platyrhynchos	20	17	4	41	35
Taeniopygia guttata	27	13	14	54	45
**Total**	**140**	**87**	**91**	**318**	**253**

Supplementary_Table 3. **Hatching times and estimates of the corresponding incubation days at which phalanx forming takes place** (i.e. the stages equivalent to HH stages 28–36 HH). The estimation of the duration of phalanx formation is variable as is the incubation time in most animals studied. At chicken stage 28 the limb is formed as a paddle and at stage 36 the first indications of the claw are present. At stage 26 the first *Sox9* expression is seen in the developing digits and in stage 36 the last *Sox9* expression is seen in the phalanx forming region in the digits tips. In all studied animals the digit pattern is assembled during this developmental period (de Bakker, Fowler et al. 2013, Montero, Lorda-Diez et al. 2017, de Bakker, van der Vos et al. 2021). All the species we studied hatch at different times from 14 days in zebra finches to more than 100 days in the Nile crocodile, see above table. From the species in the above table only the zebra finch is precocial but the extant of development times in the studied species is still significant different. The developmental phenotypes of the limbs however are comparable (figures 2 and 3, sup. Figures 1, 2 and 3) as are the expression patterns we detected. Also, with exception of the zebra finch, the hatching times and phalanx development times for each studied species are variable. Seen this variation in hatching time within and between species a general time estimate for the development of each phalanx will be always be very imprecise.

**Scientific name**	**Common name**	**Hatching time (days)**	**phalanx forming period (days)**	**Reference**
Taeniopygia guttata	zebra finch	14	2.25	(Murray, Varian-Ramos et al. 2013)
Gallus gallus	chicken	20–22	4–5	(Hamburger and Hamilton 1951)
Dromaius novaehollandiae	emu	50–56	11–12	(Nagai, Mak et al. 2011) Estimate of their figure 2
Struthio camelus	ostrich	39–42	7–8	(Gefen and Ar 2001)
Caiman latirostris	broad-snouted caiman	70–85	13–17	(Iungman, Pina and Siroski 2008)
Crocodylus niloticus	Nile crocodile	74–103	9–11	(Peterka, Sire et al. 2010)
Pelodiscus sinensis	Chinese soft-shelled turtle	45–50	14–16	(Tokita and Kuratani 2001)
Pogona vitticeps	Central bearded dragon	59–67	15–20	(Melville, Hunjan et al. 2016, Whiteley, Holleley et al. 2017)

Supplementary_Table 2 **NCBI Accession numbers of probes used.** We have deposited the sequences in the NCBI (National Center for Biotechnology Information) database.

**Scientific name**	***Bambi***	***Sox9***	***Gdf5***
Mus musculus	KX896990		KX688160
Pogona vitticeps	KX896989	MF185833	KX688159
Gekko gecko	OK338007	OK338009	OK338008
Pelodiscus sinensis	KX896991	MF185834	KX688158
Crocodylus niloticus	KX896988	JQ717196	KX688157
Gallus gallus	KX896988		KX688154
Dromaius novaehollandiae		JQ717195	

Supplementary_Table 3 **Probes used to detect possible oscillating genes in the chicken hindlimb** One *in situ* hybridisation is staining one pair of fore- and hind limbs, left or right. Contralateral limbs of the same individual have been used to visualise expression patterns of two different genes, therefore the total of embryos is less than the total of expression studies.

**Gene**	**Accession number Found by BLAST**	**Number of *in situ* hybridisation experiments**	**Expression**
ACKR3 (CXCR7)	NM_001083362.2	21	**PFR oscillating** (Figure 6)
BMP4	NM_205237.4	2	Interdigital
BMPR1B	NM_205237.4	10	PFR
DKK2	XM_015276579.4	3	Not in PFR
FGF8	U55189.1	11	PFR not oscillating
FGF10	NM_204696.2	9	PFR not oscillating
GLI1	U60762.1	2	Not in PFR
HAND1	NM_204965.2	4	Entire digit margin
HES1	XM_040679737.2	27	PFR not oscillating
HES4	NM_001005848.3	28	PFR
ID4	NM_001396104	9	PFR
MSX1	KX897001	11	Entire digit margin shift to PFR at stage 36
MSX2	NM_204559.2	2	Entire digit margin shift to PFR at stage 36
PITX1	NM_001167686.2	3	Interphalangeal joints
RELN	NM_001305123.2	14	29 AER 30-34 distal in III and IV 35-36 PFR
TFAP2B	NM_204895.2	11	Interdigital, weak
TBX3	NM_001270878.2	5	Interdigital
SULF2	XM_040688668.2	4	Interphalangeal joints
WNT5A	KX897002	9	Entire digit margin
WNT9A	NM_204981.3	25	**PFR oscillating** Figure 6)
ZEB2	NM_001318466.2	3	Interdigital
**Total**		**214**	

Supplementary_Table 6 **Correlations of the *Gdf5* intensity measurement in the developing digits**. The red numbers are the correlations between the *Gdf5* patterns in isomorphic digits.

	**I-II**	**I-III**	**I-IV**	**I-V**	**II-III**	**II-IV**	**II-V**	**III-IV**	**III-V**	**IV-V**
Pogona vitticeps **forelimb**	0.37	0.30	0.22	0.27	0.47	0.37	0.82	0.39	0.41	0.37
**hindlimb**	0.43	0.13	0.19	0.11	0.36	0.15	0.27	0.39	0.78	0.40
Gallus gallus **forelimb**					0.38	0.79	0.33			
**hindlimb**	-0.08	0.26	0.40		0.49	0.36		0.42		

Supplementary Figures

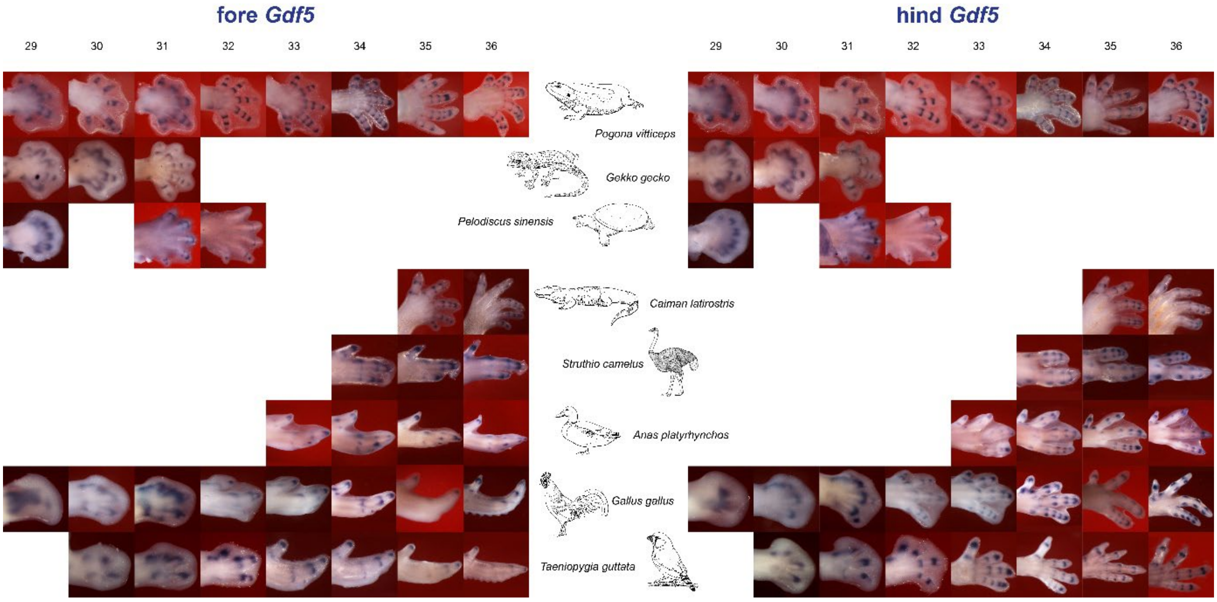

Supplementary_Figure 1 **Expression of *Gdf5* in developmental series of amniote forelimbs and hindlimbs**. For easier comparison, the images of left limbs are inverted. Anterior is to the top and distal to the right. Line drawings by Esmée Winkel.

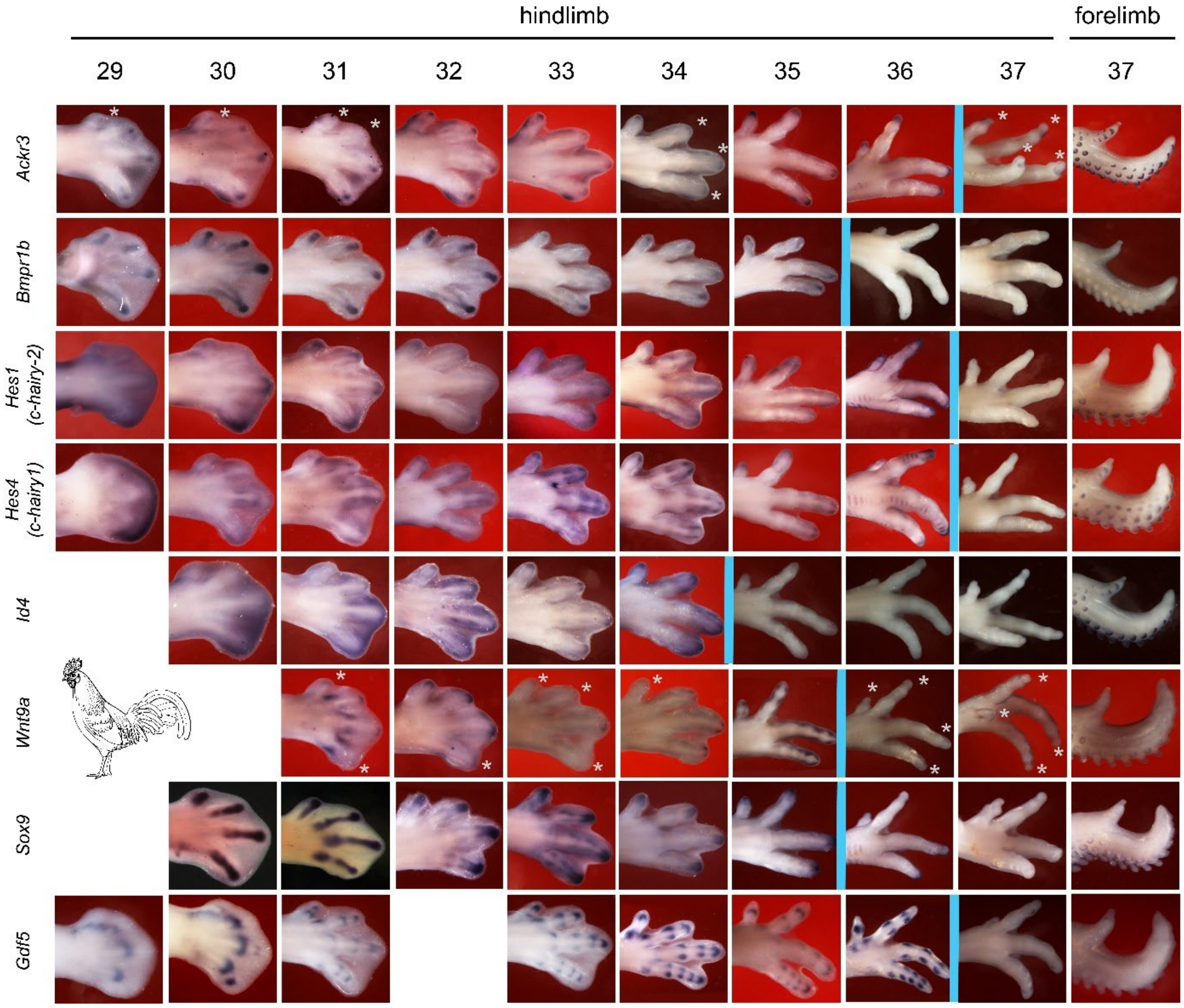

Supplementary_Figure 2 **Gene expression patterns in the developing phalanx forming region of the chicken hindlimb and forelimb.** Asterisks (*) indicate digit tips with no expression. Anterior is to the top and distal to the right. Numbers indicate HH stages. The lack of expression in the stage 37 hindlimbs is not due to a technical failure because the wing from the same embryo does show hybridisation in the feather follicles (far right column). The blue vertical lines mark the stage at which expression in the toe tips is down regulated. Ackr3 and Wnt9a expression is also shown in Figure 6.

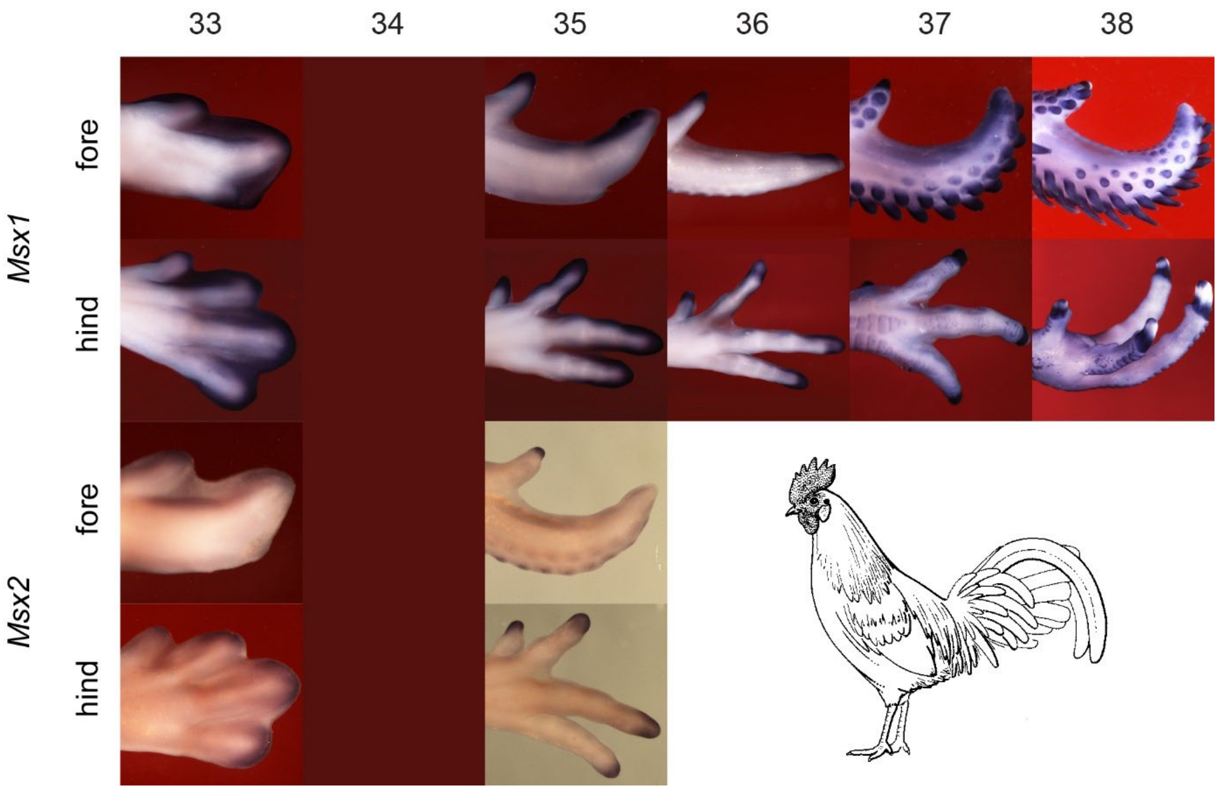

Supplementary_Figure 3 **Expression of *Msx1* and *Msx2* in the fore- and hindlimb of the chicken (*Gallus gallus*)**. Anterior to the top and distal to the right. The expression of both genes concentrates at the digit tips at stage 36 for *Msx1* and 35 for *Msx2* for the digits which will form claws, only digit II for the wing and all digits in the hindlimb. Note that both genes are also expressed in the feather primordia.

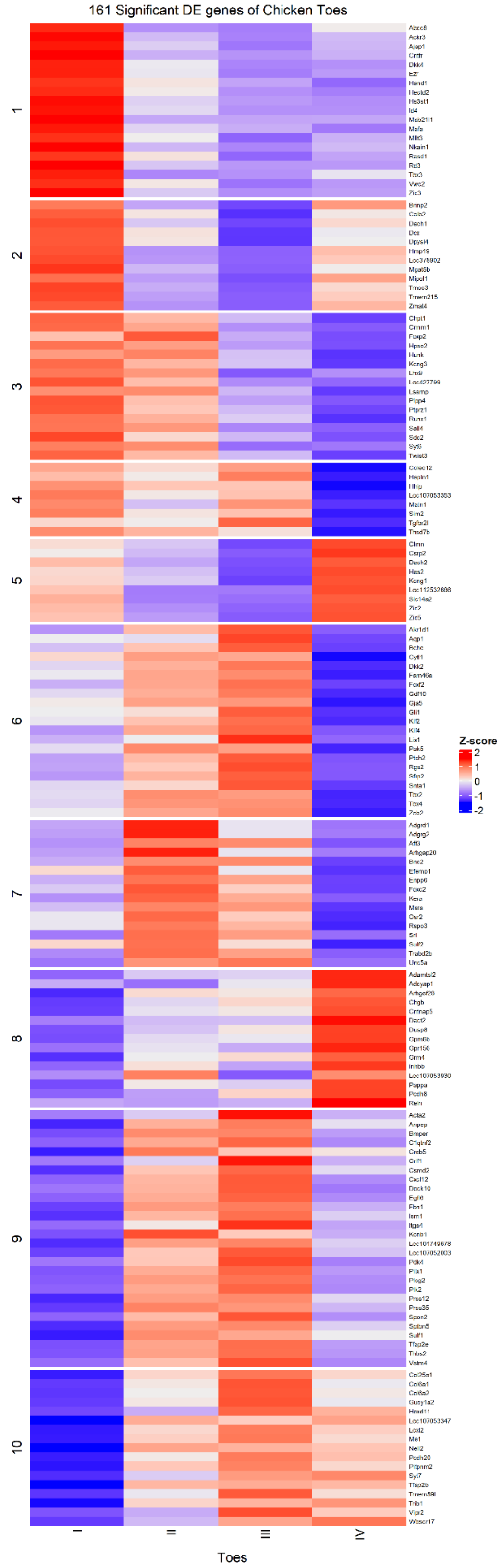

Supplementary_Figure 4 **Heatmap of 161 genes differentially expressed between the phalanx forming regions of digits I-IV of the chicken hindlimb (stage 35).**

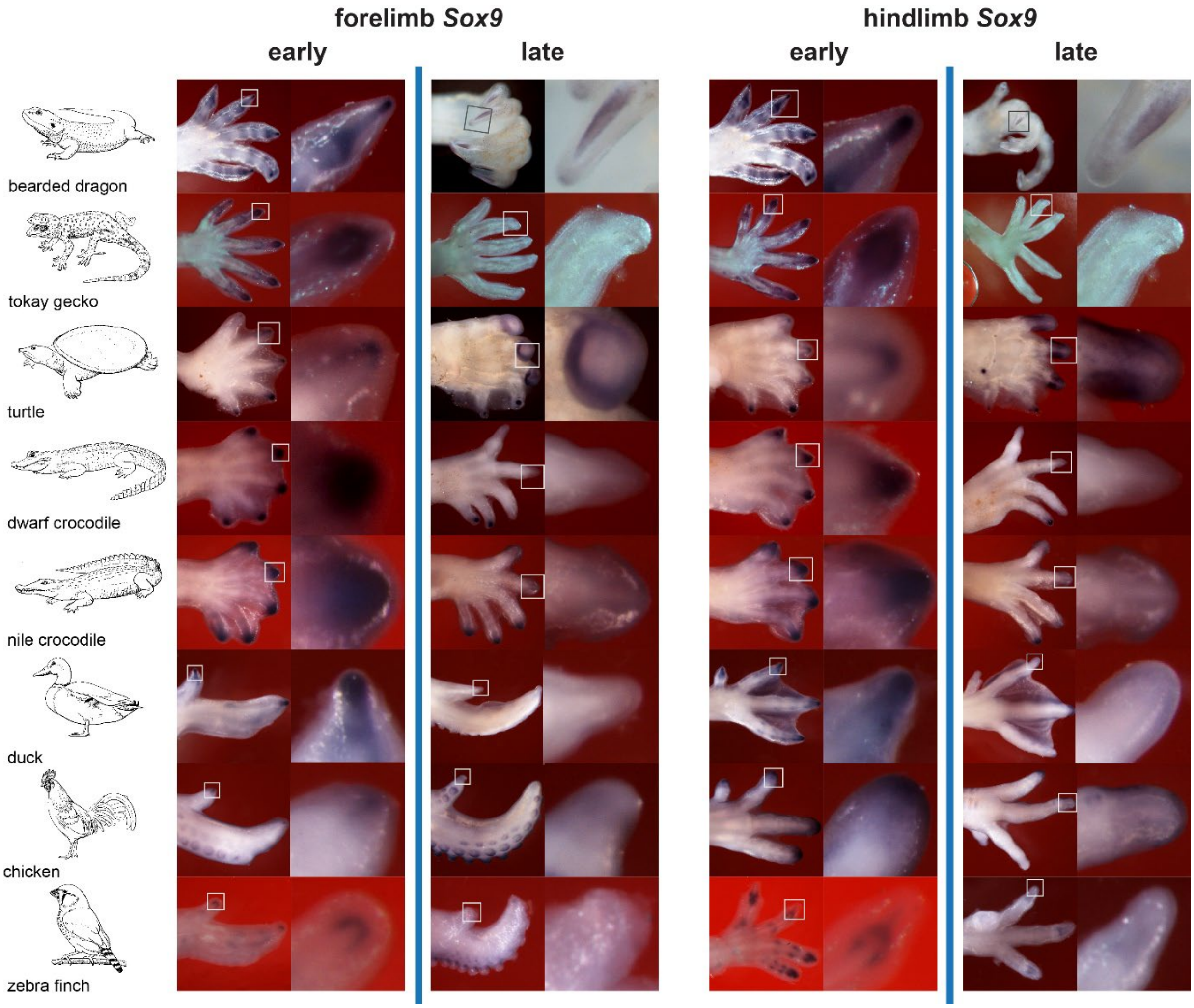

Supplementary_Figure 5 **Expression of *Sox9* is down-regulated in the phalanx forming region of the developing limbs after the phalanx-claw transition.** We show early stage limbs before the phalanx-claw transition, and late stage limbs after that transition. In the early-stage autopods *Sox9* is still expressed in the digit tips, while no expression is detected in the late-stage digit tips. The exceptions are the clawless posterior digits of the turtle and the two crocodilians, which continue to express *Sox9* at the later stage shown. Note that *Sox9* is also expressed in prechondrocytes (e.g. in the early bearded dragon) and the nail bed (late bearded dragon and turtle). For clarity we show on the right magnifications of the tips of

digits II marked with a square. For easier comparison, images of left limbs are inverted. Anterior is to the top and distal to the right. line drawings by Esmée Winkel.

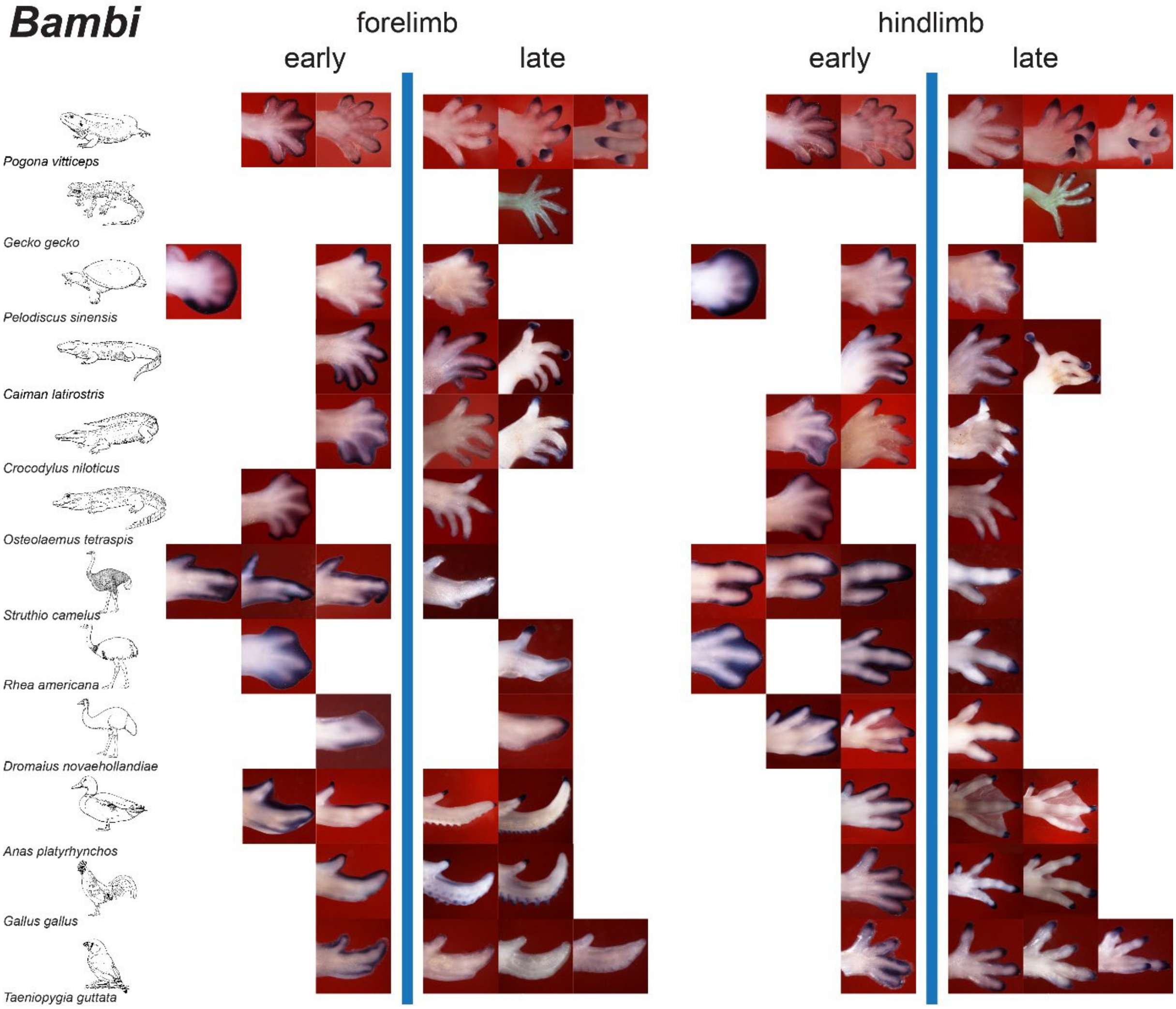

Supplementary_Figure 6 **Expression of *Bambi* in lizards, turtle, crocodiles and birds.** In the early stages *Bambi* is expressed at the margin of the whole autopod. In the late stage *Bambi* is only expressed in the digit tips which will develop a claw (see Figure 4 and main text). For easier comparison, images of left limbs are inverted. Anterior is to the top and distal to the right. Line drawings by Esmée Winkel.

Supplementary information

Supplementary_Information 1. References for the 38 significant differential expressed genes, above 25 TPM, of chicken toes stage 35

The names we use are the same as used in Gene Cards (https://www.genecards.org/). With these gene names we searched the literature on Web of Science (https://www.webofscience.com/wos/woscc/basic-search) and PubMed (https://pubmed.ncbi.nlm.nih.gov/) with the addition of limb or chick. For data on gene expression in chick and mouse we also searched Geisha (http://geisha.arizona.edu/geisha/index.jsp) and MGI (https://www.informatics.jax.org/expression.shtml)

Bold gene names directly connected to digit development according to the literature

Italic gene names no connection with digit development according to the literature

Group 1: Significant higher expression in digit I

Ackr3 (Atypical Chemokine Receptor 3 or CXCR7) This gene encodes a member of the G-protein coupled receptor family. It is among others involved in osteo-differentiation [1], identified as a senescence marker [2], and a regulator of the oscillation of glucocorticoid [3]. It is expressed in stage 28 in the metatarsophalangeal joint and from that stage till stage 36 in all the toe tips and in the interdigital tissue.

Calb2 Is a known neural marker but also expressed in the mouse limb and it is downstream to *Lmx1b* which controls the dorsal-ventral patterning of the limb [4,5]

Cntfr Is apro-inflammatory gene and belongs to IL6 family and is essential in inflammatory response, metabolism and tissue regeneration[6]. No connections to limb development found.

Dkk4 (Dickkopf WNT Signaling Pathway Inhibitor 4) is involved in the development of mouse claws[7].

Dpysl4 (Dihydropyrimidinase-related protein 4) is a known regulator of hippocampal neuron development. It is also involved in growth regulation, polarization and differentiation of dental epithelial cells during tooth germ morphogenesis[8]. No connections to limb development found.

Hand1 (Heart And Neural Crest Derivatives Expressed 1) The protein encoded by this gene belongs to the basic helix-loop-helix family of transcription factors. It is involved in limb development in mice [9,10]

Nsg2 (Neuronal Vesicle Trafficking Associated 2 also known as Hmp19) NCBI: Predicted to enable clathrin light chain binding activity. Predicted to be involved in clathrin coat assembly and endosomal transport all cellular processes. But clear expression in the embryonic mouse hindlimb (MGI).

Id4 (Inhibitor Of DNA Binding) is involved in the circadian clock, osteoblast differentiation and is expressed in the early chicken embryo (till about stage 19 HH) [11-13]. In chicken stage 34 HH foot it is expressed in all four the toe tips but not any more in stages 35-36-37 hindlimbs. It is however still expressed in the feather buds of the wings.

Mab21l1 (Mab-21 Like 1) Is expressed during mouse and bat autopod development[14,15].

Rasd1 (Ras Related Dexamethasone Induced 1) Small GTPase which is expressed in a variety of tissues including heart, brain, placenta, lung, liver, skeletal muscle, kidney and pancreas, with the strongest expression in the heart[16]. No connections to limb development found.

Tbx3 (T-Box Transcription Factor 3) this protein is a transcriptional repressor and is thought to play a role in the anterior/posterior axis of the tetrapod forelimb. Our transcriptome results (stage 35 HH) show that *Tbx3* is higher expressed anterior in the interdigital tissue while *in situ* hybridisation results in younger embryos both anterior and posterior expression or only posterior expression [14,17,18].

Group 2: Significant lower expression in digit IV

Adgrg2 (Adhesion G Protein-Coupled Receptor G2) NCBI This gene encodes a member of the G protein-coupled receptor family described as an epididymis-specific transmembrane protein. MGI light expression in mouse digits.

Cytl1 (Cytokine Like 1) involved in bone development [19,20] MGI strong expression in developing skeleton including digits.

Dkk2 (Dickkopf WNT Signalling for somePathway Inhibitor 2) member of the dickkopf family, a secreted antagonist of the Wnt signalling pathway and expressed during mouse limb development [21].

Efemp1 (EGF Containing Fibulin Extracellular Matrix Protein 1) Implicated with carpal tunnel syndrome[22] MGI expression in digit tips

Hapln1 (Hyaluronan And Proteoglycan Link Protein 1) is associated with joint development in the digits[23]

Hpse2 (Heparanase 2 (Inactive)) UniProtKB/Swiss-Prot: Binds heparin and heparan sulfate with high affinity, but lacks heparanase activity. Noggin binds strongly to heparin sulfate and is part of the Turing mechanism of phalanx development as proposed by Gall et all[24]

Hunk (Hormonally Up-Regulated Neu-Associated Kinase) NCBI Predicted to enable protein serine/threonine kinase activity. Predicted to be involved in intracellular signal transduction and protein phosphorylation. No connections to limb development found.

Kcng3 **(**Potassium Voltage-Gated Channel Modifier Subfamily G Member 3 also known as Kv6.3) A voltage-gated K^+ channel highly expressed in the brain. Homomultimeric Kv6.3 did not resulted in a functional voltage gated channel. No connections to limb development found

Iftim2 Loc107053353 (Iftim2) chIFITM2 (putative LOC107053353-dispanin-2b-like)[25] Interferon inducible transmembrane (IFITM) proteins are effectors of the immune system widely involved in restricting entry into cells of a broad range of viruses (Bassano, Ong et al. 2017 No connections to limb development found.

Matn1 (Matrilin 1) Matn1 is an extracellular matrix protein and is almost exclusively expressed in columnar and prehypertrophic chondrocytes[26]. MGI strong expression in all skeleton including digits. Matn1 is strongly expressed in the phalanges during development[23].

Osr2 (Odd-Skipped Related Transciption Factor 2) Involved in joint differntiation[27-29]

Rspo3 (R-Spondin 3) are secreted ligands that bind cell surface receptors and activate Wnt/b-catenin signalling. Knockout Rspo3 is lethal in mice and is part of the Wnt singing essential for limb development [30,31]

Sulf2 (Sulfatase 2) Heparan sulfate 6-O-endosulfatases, such as SULF2, selectively remove 6-O-sulfate groups from heparan sulfate. Both Sulf1 and Sulf2 are involved in pattering the developing mouse limb [32]. Could be partly due to the binding of Noggin to heparan sulfate, part of the Turning mechanism proposed by Grall et all[24]. The expression of *Sulf2* is in the future joints our data.

Twist3 (Twist Family BHLH Transcription Factor 3). Is involved in limb development but no very specific expressed[33,34]

Zeb2 (Zinc Finger E-Box Binding Homeobox 2) The protein encoded by this gene is a member of the Zfh1 family of 2-handed zinc finger/homeodomain proteins. It is located in the nucleus and functions as a DNA-binding transcriptional repressor that interacts with activated SMADs. The gene (Zeb2 = ZFHX1B) is expressed in the chicken and mouse hindlimb with not very clear results in the chicken [35]. Also our own gene expression data is not very convincing.

Group 3: Significant higher expression in digit IV

Adcyap1 (Adenylate Cyclase Activating Polypeptide 1) also known as Pituitary Adenylate Cyclase-Activating Polypeptide (PACAP) is a neuropeptide[36]. No connections to limb development found.

Chgb (Chromogranin B) is a neuroendocrine secretory granule protein (UniProtKB/Swiss-Prot in GeneCards) No connections to limb development found.

Csrp2 (Cysteine And Glycine Rich Protein 2) During limb regeneration in the Chinese fire-bellied newt (*Cynops orientalis*) *Crsp2* expression peaks when the autopod gets formed [37]. But not very specific in chicken [37,38]

Gpm6b (Glycoprotein M6B or Neuronal membrane glycoprotein gene 6b) the protein of this gene is a membrane glycoprotein and is upregulated during osteoblast differntation[39]

Group 4: Significant higher expression digit III

Akr1d1 (Aldo-Keto Reductase Family 1 Member D1) The enzyme encoded by this gene is responsible for the catalysis of the 5-beta-reduction of bile acid intermediates and steroid hormones carrying a delta(4)-3-one structure. No connections to limb development found.

Crlf1 **(**Cytokine Receptor Like Factor 1) UniProtKB/Swiss-Prot Summary: In complex with CLCF1 it forms a heterodimeric neurotropic cytokine that plays a crucial role during neuronal development (Probable) No connections to limb development found

Cxcl12 (C-X-C Motif Chemokine Ligand 12) is strongly involved in pattering connective tissue during chicken limb development [27,40]

Egfl6 (EGF Like Domain Multiple 6) Egfl6 (= Maeg) is expressed in the mouse digits[41]

Nell2 (Neural EGFL Like 2) is expressed in the developing mouse digits[42]

Pitx1 (Paired Like Homeodomain 1) Pitx1 is necessary for hindlimb development[43,44]

Rgs2 **(**Regulator Of G Protein Signalling 2) Regulator of G protein signalling (RGS) family members are regulatory molecules that act as GTPase activating proteins (GAPs) for G alpha subunits of heterotrimeric G proteins. No connections to limb development found

Sulf1 (Sulfatase 1) Is expressed during joint formation [32,45,46]

From the literature we studied several other genes involved in the timing of gene expression and/or limb development: *Bmpr1b* [47]; *Hes1*(*c-Hairy2*)[48-51]; *Hes4* (*c-Hairy1*)[48,52]

Supplementary References

1 Liu, J., Yao, X. T., Feng, X. L. & Bai, X. Z. BMP2 induces osteogenic differentiation through ACKR3 in mesenchymal stem cells. *Biochem Biophys Res Commun* **664**, 59-68, doi:10.1016/j.bbrc.2023.04.097 (2023).

2 Takaya, K., Asou, T. & Kishi, K. Selective Elimination of Senescent Fibroblasts by Targeting the Cell Surface Protein ACKR3. *Int J Mol Sci* **23**, doi:10.3390/ijms23126531 (2022).

3 Quinn, K. E., Mackie, D. I. & Caron, K. M. Emerging roles of atypical chemokine receptor 3 (ACKR3) in normal development and physiology. *Cytokine* **109**, 17-23, doi:10.1016/j.cyto.2018.02.024 (2018).
4 Hoekstra, E. J. *et al.* Lmx1a encodes a rostral set of mesodiencephalic dopaminergic neurons marked by the Wnt/B-catenin signaling activator R-spondin 2. *PLoS One* **8**, e74049, doi:10.1371/journal.pone.0074049 (2013).
5 Krawchuk, D. & Kania, A. Identification of genes controlled by LMX1B in the developing mouse limb bud. *Dev Dyn* **237**, 1183-1192, doi:10.1002/dvdy.21514 (2008).
6 Nan, J. *et al.* Transcriptome analysis of multiple tissues reveals the potential mechanism of death under acute heat stress in chicken. *BMC Genomics* **24**, 459, doi:10.1186/s12864-023-09564-2 (2023).
7 Cui, C. Y. *et al.* Deficiency Disrupts the Differentiation Process of Nail Development. *J Invest Dermatol* **133**, 1990-1997, doi:10.1038/jid.2013.84 (2013).
8 Yasukawa, M. *et al.* Is Involved in Tooth Germ Morphogenesis through Growth Regulation, Polarization and Differentiation of Dental Epithelial Cells. *Int J Biol Sci* **9**, 382-390, doi:10.7150/ijbs.5510 (2013).
9 Firulli, B. A. *et al.* Defective Hand1 phosphoregulation uncovers essential roles for Hand1 in limb morphogenesis. *Development* **144**, 2480-2489, doi:10.1242/dev.149963 (2017).
10 Laurie, L. E., Kokubo, H., Nakamura, M., Saga, Y. & Funato, N. The Transcription Factor Hand1 Is Involved In Runx2-Ihh-Regulated Endochondral Ossification. *Plos One* **11**, doi:ARTN e0150263

11 Duffield, G. E., Robles-Murguia, M., Hou, T. Y. & McDonald, K. A. Targeted Disruption of the Inhibitor of DNA Binding 4 (Id4) Gene Alters Photic Entrainment of the Circadian Clock. *Int J Mol Sci* **22**, doi:ARTN 9632

12 Kee, Y. & Bronner-Fraser, M. Id4 expression and its relationship to other Id genes during avian embryonic development. *Mech Dev* **109**, 341-345, doi:10.1016/s0925-4773(01)00576-7 (2001).
13 Tokuzawa, Y. *et al.* Id4, a new candidate gene for senile osteoporosis, acts as a molecular switch promoting osteoblast differentiation. *PLoS Genet* **6**, e1001019, doi:10.1371/journal.pgen.1001019 (2010).
14 Dai, M. *et al.* Differential expression of Meis2, Mab21l2 and Tbx3 during limb development associated with diversification of limb morphology in mammals. *PLoS One* **9**, e106100, doi:10.1371/journal.pone.0106100 (2014).
15 Wong, R. L., Chan, K. K. & Chow, K. L. Developmental expression of Mab21l2 during mouse embryogenesis. *Mech Dev* **87**, 185-188, doi:10.1016/s0925-4773(99)00127-6 (1999).
16 Tu, Y. & Wu, C. Cloning, expression and characterization of a novel human Ras-related protein that is regulated by glucocorticoid hormone. *Biochim Biophys Acta* **1489**, 452-456, doi:10.1016/s0167-4781(99)00197-9 (1999).
17 Fisher, M. *et al.* Comparative analysis of 3D expression patterns of transcription factor genes and digit fate maps in the developing chick wing. *PLoS One* **6**, e18661, doi:10.1371/journal.pone.0018661 (2011).
18 Tumpel, S. *et al.* Regulation of Tbx3 expression by anteroposterior signalling in vertebrate limb development. *Dev Biol* **250**, 251-262 (2002).
19 Jeon, J. *et al.* Cytokine-like 1 knock-out mice (Cytl1-/-) show normal cartilage and bone development but exhibit augmented osteoarthritic cartilage destruction. *J Biol Chem* **286**, 27206-27213, doi:10.1074/jbc.M111.218065 (2011).
20 Kim, J. S., Ryoo, Z. Y. & Chun, J. S. Cytokine-like 1 (Cytl1) regulates the chondrogenesis of mesenchymal cells. *J Biol Chem* **282**, 29359-29367, doi:10.1074/jbc.M700965200 (2007).
21 Witte, F., Dokas, J., Neuendorf, F., Mundlos, S. & Stricker, S. Comprehensive expression analysis of all Wnt genes and their major secreted antagonists during mouse limb

development and cartilage differentiation. *Gene Expr Patterns* **9**, 215-223, doi:10.1016/j.gep.2008.12.009 (2009).
22 Wiberg, A. *et al.* A genome-wide association analysis identifies 16 novel susceptibility loci for carpal tunnel syndrome. *Nat Commun* **10**, 1030, doi:10.1038/s41467-019-08993-6 (2019).
23 Nowosad, K. *et al.* Identification of candidate enhancers controlling the transcriptome during the formation of interphalangeal joints. *Sci Rep* **12**, 12835, doi:10.1038/s41598-022-16951-4 (2022).
24 Grall, E. *et al.* Self-organized BMP signaling dynamics underlie the development and evolution of digit segmentation patterns in birds and mammals. *Proc Natl Acad Sci U S A* **121**, e2304470121, doi:10.1073/pnas.2304470121 (2024).
25 Bassano, I. *et al.* Accurate characterization of the IFITM locus using MiSeq and PacBio sequencing shows genetic variation in Galliformes. *BMC Genomics* **18**, 419, doi:10.1186/s12864-017-3801-8 (2017).
26 Szénási, T. *et al.* Hmgb1 can facilitate activation of the matrilin-1 gene promoter by Sox9 and L-Sox5/Sox6 in early steps of chondrogenesis. *Bba-Gene Regul Mech* **1829**, 1075-1091, doi:10.1016/j.bbagrm.2013.07.004 (2013).
27 Orgeur, M. *et al.* Genome-wide strategies identify downstream target genes of chick connective tissue-associated transcription factors. *Development* **145**, doi:ARTN dev161208
28 Stricker, S., Brieske, N., Haupt, J. & Mundlos, S. Comparative expression pattern of Odd-skipped related genes Osr1 and Osr2 in chick embryonic development. *Gene Expr Patterns* **6**, 826-834, doi:10.1016/j.modgep.2006.02.003 (2006).
29 Yeboah, R. L. *et al.* Sox, Fox, and Lmx1b binding sites differentially regulate a Gdf5-Associated regulatory region during elbow development. *Front Cell Dev Biol* **11**, 1215406, doi:10.3389/fcell.2023.1215406 (2023).
30 Neufeld, S. *et al.* A conditional allele of Rspo3 reveals redundant function of R-spondins during mouse limb development. *Genesis* **50**, 741-749, doi:10.1002/dvg.22040 (2012).
31 Szenker-Ravi, E. *et al.* RSPO2 inhibition of RNF43 and ZNRF3 governs limb development independently of LGR4/5/6. *Nature* **557**, 564-569, doi:10.1038/s41586-018-0118-y (2018).
32 Ratzka, A. *et al.* Redundant function of the heparan sulfate 6-O-endosulfatases Sulf1 and Sulf2 during skeletal development. *Dev Dyn* **237**, 339-353, doi:10.1002/dvdy.21423 (2008).
33 Hornik, C., Krishan, K., Yusuf, F., Scaal, M. & Brand-Saberi, B. cDermo-1 misexpression induces dense dermis, feathers, and scales. *Dev Biol* **277**, 42-50, doi:10.1016/j.ydbio.2004.08.050 (2005).
34 Scaal, M., Fuchtbauer, E. M. & Brand-Saberi, B. cDermo-1 expression indicates a role in avian skin development. *Anat Embryol (Berl)* **203**, 1-7, doi:10.1007/pl00008244 (2001).
35 Tylzanowski, P., De Valck, D., Maes, V., Peeters, J. & Luyten, F. P. Zfhx1a and Zfhx1b mRNAs have non-overlapping expression domains during chick and mouse midgestation limb development (vol 3, pg 39, 2003). *Gene Expression Patterns* **3**, 383-383, doi:10.1016/S1567-133x(03)00052-8 (2003).
36 Lepeak, L. *et al.* Pituitary Adenylate Cyclase-Activating Polypeptide (PACAP) of the Bed Nucleus of the Stria Terminalis Mediates Heavy Alcohol Drinking in Mice. *eNeuro* **10**, doi:10.1523/ENEURO.0424-23.2023 (2023).
37 Feng, Y. *et al.* Molecular cloning, characterization, and expression analysis of the three cysteine and glycine-rich protein genes in the Chinese fire-bellied newt Cynops orientalis. *Gene* **647**, 226-234, doi:10.1016/j.gene.2018.01.012 (2018).
38 Bonnin, M. A., Edom-Vovard, F., Kefalas, P. & Duprez, D. CRP2 transcript expression pattern in embryonic chick limb. *Mech Dev* **116**, 151-155, doi:10.1016/s0925-4773(02)00122-3 (2002).
39 Drabek, K., van de Peppel, J., Eijken, M. & van Leeuwen, J. P. GPM6B regulates osteoblast function and induction of mineralization by controlling cytoskeleton and matrix vesicle release. *J Bone Miner Res* **26**, 2045-2051, doi:10.1002/jbmr.435 (2011).

40 Nassari, S. *et al.* The chemokines CXCL12 and CXCL14 differentially regulate connective tissue markers during limb development. *Sci Rep* **7**, 17279, doi:10.1038/s41598-017-17490-z (2017).
41 Buchner, G. *et al.* MAEG, an EGF-repeat containing gene, is a new marker associated with dermatome specification and morphogenesis of its derivatives. *Mech Dev* **98**, 179-182, doi:10.1016/s0925-4773(00)00462-7 (2000).
42 Beccari, L. *et al.* Dbx2 regulation in limbs suggests interTAD sharing of enhancers. *Dev Dyn* **250**, 1280-1299, doi:10.1002/dvdy.303 (2021).
43 Logan, M. & Tabin, C. J. Role of Pitx1 upstream of Tbx4 in specification of hindlimb identity. *Science* **283**, 1736-1739, doi:10.1126/science.283.5408.1736 (1999).
44 Marcil, A., Dumontier, E., Chamberland, M., Camper, S. A. & Drouin, J. Pitx1 and Pitx2 are required for development of hindlimb buds. *Development* **130**, 45-55, doi:10.1242/dev.00192 (2003).
45 Wang, Y. H. & Beck, C. Distinct patterns of endosulfatase gene expression during Xenopus laevis limb development and regeneration. *Regeneration (Oxf)* **2**, 19-25, doi:10.1002/reg2.27 (2015).
46 Zhao, W., Sala-Newby, G. B. & Dhoot, G. K. Sulf1 expression pattern and its role in cartilage and joint development. *Dev Dyn* **235**, 3327-3335, doi:10.1002/dvdy.20987 (2006).
47 Baur, S. T., Mai, J. J. & Dymecki, S. M. Combinatorial signaling through BMP receptor IB and GDF5: shaping of the distal mouse limb and the genetics of distal limb diversity. *Development* **127**, 605-619 (2000).
48 Jouve, C. *et al.* Notch signalling is required for cyclic expression of the hairy-like gene HES1 in the presomitic mesoderm. *Development* **127**, 1421-1429, doi:10.1242/dev.127.7.1421 (2000).
49 Pascoal, S. *et al.* A molecular clock operates during chick autopod proximal-distal outgrowth. *J Mol Biol* **368**, 303-309 (2007).
50 Sheeba, C. J., Palmeirim, I. & Andrade, R. P. Retinoic acid signaling regulates embryonic clock hairy2 gene expression in the developing chick limb. *Biochem Bioph Res Co* **423**, 889-894 (2012).
51 Sharma, D. *et al.* HES1 is a novel downstream modifier of the SHH-GLI3 Axis in the development of preaxial polydactyly. *PLoS Genet* **17**, e1009982, doi:10.1371/journal.pgen.1009982 (2021).
52 Vasiliauskas, D., Laufer, E. & Stern, C. D. A role for hairy1 in regulating chick limb bud growth. *Dev Biol* **262**, 94-106, doi:10.1016/s0012-1606(03)00360-9 (2003).
